# Evolving Concepts of Emotion and Motivation

**DOI:** 10.3389/fpsyg.2018.01647

**Published:** 2018-09-07

**Authors:** Kent C. Berridge

**Affiliations:** Department of Psychology, University of Michigan, Ann Arbor, MI, United States

**Keywords:** reward, addiction, emotion, fear, disgust, history of psychology, psychological science, psychological method

## Abstract

This review takes a historical perspective on concepts in the psychology of motivation and emotion, and surveys recent developments, debates and applications. Old debates over emotion have recently risen again. For example, are emotions necessarily subjective feelings? Do animals have emotions? I review evidence that emotions exist as core psychological processes, which have objectively detectable features, and which can occur either with subjective feelings or without them. Evidence is offered also that studies of emotion in animals can give new insights into human emotions. Beyond emotion, motivation concepts have changed over decades too, and debates still continue. Motivation was once thought in terms of aversive drives, and reward was thought of in terms of drive reduction. Motivation-as-drive concepts were largely replaced by motivation-as-incentive concepts, yet aversive drive concepts still occasionally surface in reward neuroscience today. Among incentive concepts, incentive salience is a core motivation process, mediated by brain mesocorticolimbic systems (dopamine-related systems) and sometimes called ‘wanting’ (in quotation marks), to distinguish it from cognitive forms of desire (wanting without quotation marks). Incentive salience as ‘wanting’ is separable also from pleasure ‘liking’ for the same reward, which has important implications for several human clinical disorders. Ordinarily, incentive salience adds motivational urgency to cognitive desires, but ‘wanting’ and cognitive desires can dissociate in some conditions. Excessive incentive salience can cause addictions, in which excessive ‘wanting’ can diverge from cognitive desires. Conversely, lack of incentive salience may cause motivational forms of anhedonia in depression or schizophrenia, whereas a negatively-valenced form of ‘fearful salience’ may contribute to paranoia. Finally, negative ‘fear’ and ‘disgust’ have both partial overlap but also important neural differences.

## Introduction

This review discusses the history of motivation and emotion concepts in psychology and affective neuroscience, drawing on both animal studies and human studies, in order to gain a better perspective on recent concepts and debates. In this discussion, I will sometimes combine motivation and emotion together because those psychological categories overlap in phenomena, making it hard to draw absolute distinctions. For example, is fear a stimulus-elicited emotion or a goal-achieving motivation? Or both?

Conversely, I will often distinguish between conscious or subjective feelings and unconscious or objective core processes within emotion and motivation. Although these often occur together, they sometimes need to be considered separately. To aid that distinction I will use quotation marks around ‘wanting,’ ‘liking,’ ‘fear’ and ‘disgust’ to distinguish those as objective core processes, that can be either merely unconscious or also conscious, from accompanying always conscious feelings denoted by the same words without quotation marks. The issue of whether emotions can exist independently of subjective feelings is important enough that we should deal with it right away.

## Subjective Feelings Versus Objective Features of Motivation/Emotion

In the first half of the 20th century, to speak about subjective feelings was considered misguided by behaviorist psychologists and reductionist neuroscientists. To behaviorists and reductionists, subjective feeling measures were irrelevant, and feelings were mere epiphenomena. At best, they thought feelings were old-fashioned relics of early introspectionist psychologists, such as E.B. Titchener, who around 1900 analyzed introspective reports of sensory feelings using trained observers, but found results that were often peculiar to particular observers and in the end amounted to little of general value (Titchener, 1902).

Today subjective feelings again are of wide interest, and for some the pendulum has moved to the opposite extreme. For example, Joseph LeDoux, a distinguished affective neuroscientist, recently argued that emotion is necessarily always and only a subjective feeling, and that verbal reports of feelings are the ‘gold standard’ of evidence needed to conclude that any emotion exists (LeDoux, 2014; LeDoux J., 2015; LeDoux and Hofmann, 2018). For example, LeDoux and Hofmann write “*subjective emotional experience, the feeling, is the essence of an emotion*,” and the only reliable “*way to assess conscious emotional feelings is through verbal self-repor*t” (LeDoux and Hofmann, 2018, p. 67). For individuals who cannot speak about feelings, including animals, human infants, or brain-damaged aphasic human adults, LeDoux reverts to a stance shared with early behaviorists: such creatures are regarded to have mere reactions, without any emotional feelings at all. He suggests that “*Infants can react “emotionally” long before they can feel emotion… Similarly, it is possible, in fact likely, that animals can react “emotionally” “without feeling*” (LeDoux, 2014, p. 2876). To react without feeling is not to have a true emotion at all, according to LeDoux, and he assumes that animal reactions lack any conscious feeling : “*I thus assume, until proven otherwise, that a defensive organismic state and its constituent components are implicit (non-conscious)*” (parenthetical phrase in original) (LeDoux, 2014).

This is a striking change in position, because LeDoux is well known for earlier work that aimed to map fear in amygdala-related circuitry of rats. LeDoux considers now that the question of “*whether animals react but do not feel, or whether they both react and feel, is, in my opinion, not something we can determine scientificall*y” (LeDoux, 2014, p. 2876). To be unable to know something is to be agnostic. But LeDoux goes beyond an agnostic stance regarding animal emotions and instead adopts one of positive denial. LeDoux rejects his own former view of amygdala as a fear system, writing “*I and others have called the brain system that detects and responds to threats the fear system (in rats). This was a mistake that has led to much confusion. … I will propose and defend a different way of talking about this research, one that focuses on the actual subject matter and data (threat detection and defense response*s)” (LeDoux, 2014, p.2871). In short, LeDoux currently grants animals to have brain threat detectors and the capacity for defense responses, but neither fear as a psychological emotion nor the brain systems needed for emotional feelings.

LeDoux notes that his view is similar to the dictionary definition of emotion as a subjective feeling. He is also not alone among modern psychologists in insisting that emotions must always be conscious (Clore, 1994), nor in denying emotional feelings to animals (Barrett, 2017). For example, the prominent psychologist Lisa Feldman-Barrett in her recent book on emotion raises the question ‘*does a growling dog feel anger?*’, and answers “*The answer is almost certainly no. Dogs do not have the human emotion concepts necessary to construct an instance of anger*” (Barrett, 2017, p. 269). This rejection of animal emotion resembles LeDoux’s, but is on different, more cognitive, grounds. In Feldman-Barrett’s view emotion requires the complex cognitive appraisals, language-based reasoning and sociocultural construals of situations and meaning that only humans possess. This position continues a long tradition of earlier cognitive appraisal theories that reinterpreted emotions as essentially just another type of cognition, turning emotions essentially into cultural-linguistic representations of semantic meaning (Arnold, 1960; Lazarus, 1981; Clore and Ortony, 1984; Ellsworth, 1994; Russell and Barrett, 1999; Ellsworth and Scherer, 2003). Cognitivist academics focus on reasoning and language, and place such a high premium on rationality that they are inclined to see all psychological processes through a purely cognitive lens.

Regarding consciousness, older versions exist in psychology for the notion that emotions must be subjectively felt. The early 1900s position of Sigmund Freud, despite his reputation as father of the psychological unconscious, presaged the modern assertion that emotions are always and necessarily felt. Although Freud held that many psychological processes could be unconscious, he asserted that emotions in particular must always be conscious. Posing the question, “*are there also unconscious instinctual impulses, emotions and feelings…?”*, Freud answers himself no, because “*for emotions, feelings and affects to be unconscious would be quite out of the question*” (Freud, 1950, pp. 109–110). Freud did not go on to explain particular reasons for why he believed emotions must always be conscious, when he granted that cognitions, memories and perceptions could all occur in unconscious forms. But after all, Freud’s patients were paying him to alleviate their conscious emotional distress.

On the other hand, many contemporary psychologists and affective neuroscientists do believe that affective reactions and emotions can occur unconsciously as implicit processes, as well as subjective feelings (Zajonc, 1980; Morris et al., 1998; Damasio, 2004; Winkielman et al., 2005; Anderson and Adolphs, 2014; Berridge and Kringelbach, 2015; Frijda, 2016; Greenwald and Banaji, 2017). Many have similarly argued that emotions are shared with animals, and that emotional brain systems arose early in evolution, well before human cognitive abilities such as language-based cognition (James, 1884; Solomon and Corbit, 1974; Zajonc, 1980; Ekman, 1999; Damasio, 2004; Winkielman et al., 2005; Frijda, 2007; Frijda and Parrott, 2011; Panksepp, 2011; Anderson and Adolphs, 2014; Berridge and Kringelbach, 2015). These views are very well expressed by the distinguished affective neuroscientist Antonio Damasio in a recent book on affect, emotions and feelings (Damasio, 2018). On whether brain systems for emotional feelings are shared with animals, Damasio writes, “*Moreover, the emergence of subjectivity is not recent at all, let alone exclusively human. It is likely to have happened long ago, over the Cambrian period” (p. 238).* That is, by his view the necessary neural circuitry first evolved several 100 million years ago, being embedded in ancient wiring patterns that are largely contained in deep brain structures below the cortex (Swanson, 2005; Strausfeld and Hirth, 2013). And regarding the need to conceptually distinguish between feelings and emotions, despite their overlap in everyday experience, Damasio suggests, “*the felt experiences of emotions are unfortunately known by exactly the same name as the emotions themselves. This has helped perpetuate the notion that emotions and feelings are one and the same phenomenon, although they are quite distinc*t” (Damasio, 2018, p. 100).

My own view is similar: that affective reactions can occur unconsciously, as well as consciously, and that we share many emotional processes and their brain circuitry with animals. But assertions, pro or con, are only an entry point to this discussion. We need reasons and evidence to form adequate conclusions. What is the actual evidence regarding emotions? Is it an adequate theory for psychology to say that animals don’t have emotions or that emotions must be conscious?

Quite a lot of evidence actually contradicts contentions that emotions are always subjective feelings, and instead suggests that emotions can also occur even in people without being subjectively felt – at least under some conditions. These emotional reactions occur as core affective processes that can remain intrinsically psychological and emotional, even without conscious feelings, and which have objective consequences and features that can be detected in physiology and/or behavior. However, before sketching that evidence, I should point out one important point of agreement between my view and those of Damasio and LeDoux, despite differences on whether emotion exists in animals or is always necessarily conscious. We all agree it is important to distinguish between subjective emotional feelings and objective emotional reactions. The difference has important implications for understanding normal psychological processes and for understanding psychological disorders.

### Avoiding Double-Standards for Proof

Perhaps the main reason LeDoux gives for denying emotions to animals or infants is that we cannot know with scientific certainty that they have subjective feelings: we have no proof of what they feel. That objection has merit, in the sense that we do lack proof and scientific certainty about conscious feelings of animals and infants. But proof and certainty are the key words here, and we should be careful to avoid double standards in using them. We also lack proof and scientific certainty about the subjective experience of every other human adult besides ourselves. Can I be scientifically certain about your subjective feelings? Can you say with scientific certainty whether your fear feels the same as mine? Do I know with certainty when you are happy or sad? Certainty is not the form of knowledge we have about subjective experience in any other mind than our own (Nagel, 1974).

Yet LeDoux is willing to accept verbal reports from fellow adult humans about feelings as evidence of emotion on the grounds (1) that “*If my brain can be conscious, so can yours” and (2) “because our species is naturally endowed with language, we can share…the amazing sight of the sun setting over the ocean*” (LeDoux J., 2015, p. 49). All of us might agree. But this is a socially convenient agreement based on mutual empathy. It is essentially an assumption we are willing to make for each other (and perhaps evolutionarily compelled to make), but not a scientific certainty. It is mere conjecture to say that only other humans have the crucial brain similarity needed for consciousness but that animals do not. Clearly there is no scientific certainty about the minimum neural circuitry needed for consciousness: neuroscientists disagree among themselves over whether the essential basis is cortical versus subcortical, and so on. Dropping a demand for scientific certainty for emotion in other humans, but insisting on it for animals, is to adopt a double standard on proof. It is entirely understandable that we discard demands for scientific certainty in social interactions with other people. But let’s not delude ourselves that we are being consistent about scientific certainty if we apply higher standards to animals. And let’s not apply a double standard – there’s nothing scientific about that.

However, even most science is not about proof in the strong sense of certainty. Indeed, some philosophers of science have argued that scientific data never fully prove any hypothesis with complete certainty (there is always a potential alternative lurking in the wings), but only can falsify bad hypotheses (Popper, 1972). In fact, most science is rather a matter of reducing uncertainty by degrees, experiment by experiment, gradually building incremental evidence for a particular hypothesis, and gradually ruling out specific alternatives. Uncertainty almost never goes away completely. Even successful hypotheses remain forever vulnerable to being challenged and replaced. Demanding certainty before taking a hypothesis seriously would stop a lot of valuable science in its tracks.

### Resolving Whether Emotions Are Necessarily Feelings

So if scientific certainty is not the way to decide about emotions, what is? Three levels of consideration seem to me relevant in deciding the question of whether emotions are exclusively conscious: (1) *a priori* definition, (2) available evidence on unconscious emotions, and (3) applications from conclusions based on emotional studies in animals to understanding human emotions and psychological disorders.

First, *definition*: it is perfectly legitimate to say “By emotion, I mean a conscious feeling.” That’s just a matter of personal word use. But that doesn’t tell us anything about the essence of emotion as psychological fact. To go on to claim that emotion as a psychological process is exclusively a conscious feeling is to make an empirical claim about psychological fact. This claim requires actual evidence, which must be looked for in psychological studies. No one can define facts in advance.

Second, what is the *evidence?* Quite a lot of evidence exists, and in my view it does not support the idea that emotions are necessarily conscious, nor that verbal reports are the best way to assess emotion. Considerable experimental evidence indicates that some features of human emotion and motivation cannot actually be accessed well via introspection or described in subjective reports (Nisbett and Wilson, 1978; Wilson and Schooler, 1991; Schwarz, 1999; Dijksterhuis et al., 2006; Schooler and Mauss, 2010; Greenwald and Banaji, 2017; Torre and Lieberman, 2018).

In some cases, asking people to describe their emotional reasons for making a choice may lead them to construct false explanations of their own behavior. It can lead them astray from their immediate gut reactions that would be more emotionally authentic. Thinking too much and trying to verbally report feelings can distort emotional reactions. Indeed, bringing introspective attention to pleasure feelings may actually dissipate those hedonic feelings: less introspective attention can mean more emotion, as well as more accuracy about the underlying emotional reaction, in some cases (Wilson and Schooler, 1991; Dijksterhuis et al., 2006; Schooler and Mauss, 2010; Torre and Lieberman, 2018).

Studies of implicit prejudice similarly suggest that introspective verbal reports may miss some important emotional reactions (Greenwald and Banaji, 2017). Implicit prejudices can only be revealed by objective measures, such as the emotional Stroop test of reaction time to affective mismatch, sometimes to the surprise and dismay of the person who is subjectively unprejudiced. Writing about implicit prejudices, the distinguished psychologists Anthony Greenwald and Mahzarin Banaji conclude, “*When people attempt to report on their conscious perceptions and judgments, they do so not based on valid introspection but by using traces of past (possibly biased) experience to construct (possibly invalid) theories of current data*.” (italics and parenthetical phrases in original; Greenwald and Banaji, 2017, p. 868). What is subjectively reported may only be a constructed explanation of what we think we should feel or would like to feel, rather than an accurate readout of underlying emotional reactions. In other words, introspective reports can be very far from ‘gold standard’ evidence about underlying emotional processes.

There is also evidence that basic affective reactions can be triggered unconsciously by subliminal stimuli in ordinary humans, and can remain unreportable as feelings even when they go on to change a person’s behavior and subsequent judgments (Zajonc, 1980; Fischman and Foltin, 1992; Berridge and Winkielman, 2003; Winkielman and Berridge, 2004; Winkielman et al., 2005). For example, cocaine addicts under some conditions will objectively work to take cocaine infusions, and choose them over saline infusions, even when the cocaine dose is too low to produce any subjective drug feelings of pleasure or arousal (Fischman and Foltin, 1992). Further, in ordinary adults under certain circumstances, subliminally brief visual flashes of happy or angry facial expressions can elicit unconscious affective reactions, either positive or negative, which produced no change in subjective mood reports but nonetheless controlled motivated behavior some minutes afterward (Winkielman et al., 2005). In that study, before and after seeing subliminal faces, participants rated their emotional feelings. Their subjective ratings of mood or emotion were not changed at all by exposures to subliminal and backward-masked emotional faces, whether happy or angry (Winkielman et al., 2005). Yet subliminal exposure to happy faces elicited a positive affective reaction that remained unconscious, but which could be revealed by presenting thirsty participants with a relevant incentive: a pitcher of fruit beverage. Those happy-exposed participants poured more drink, drank more of what they poured, and were willing to pay a much higher monetary price for the drink offered than after viewing emotionally-neutral faces. It might be objected that subliminally viewing a happy facial expression merely increased unvalenced arousal, which increased drink motivation, and was not truly affective. However, the test of true affect is whether it is valenced positive versus negative. Valence was evident in that subliminal viewing of angry faces had an opposite negative-valenced impact on the same thirsty participants: they poured less, drank less, and were willing to pay less than after seeing only neutral facial expressions – again without any intervening change in their subjective ratings of emotional mood (Berridge and Winkielman, 2003; Winkielman et al., 2005).

In passing, it is important that these unconscious affects were evident only if participants were already thirsty (Winkielman et al., 2005). Thirst is a physiological state that should specifically amplify brain mesolimbic reactivity to perception of a drink, amplifying its incentive salience, and providing a higher brain motivation signal for unconscious emotional reactions to modulate. Psychologically, thirst combined with unconscious affective reactions were able to modulate the incentive salience of the drink in a valenced up-or-down fashion– making it more ‘wanted’ after happy faces, and less ‘wanted’ than usual after angry faces. This interaction between percept and state in controlling motivational value follows what I call Bindra-Toates rules of incentive salience, which will be described below. Of course, subliminally seeing emotional facial expressions is not equivalent to inducing the corresponding full emotion (Adolphs et al., 2005; Anderson et al., 2006). But subliminal expressions do induce a valenced affective reaction in human viewers: positive to smiling faces and negative to angry faces (Zajonc, 1980). For now, my main point is simply that these results demonstrate that bivalent affective reactions can be subjectively unconscious even in adult humans, and even when detected objectively in behavior. Defining affect or emotion solely as subjective feelings misses something important.

Finally, regarding language as a gold standard for emotion, talking about feelings evoked by sunsets is not proof even of the emotions that are declared. Linguistic computer programs may soon pass an emotional Turing test by talking compellingly about enjoyment of sunsets, so that a listener thinks the computer has emotional feelings. Even in the 1970s some people poured out their own feelings to ELIZA, a linguistic computer program with scripted responses that simulated the responsive questions of a Rogerian psychoanalyst via text messages, and seemed to human interlocutors to convey great emotional sensitivity (e.g., Client typed: “It’s true. I am unhappy.” *ELIZA responded: Do you think coming here will help you not to be unhappy?* Client: “I need some help, that much seems certain.” *ELIZA: What would it mean to you if you got some help?* Client: “Perhaps I could learn to get along with my mother.” *ELIZA: Tell me more about your family*.) (Weizenbaum, 1977). Modern artificial intelligence is creating robotic companions with linguistic scripts that are far more compelling and flexible, able to talk with their human owners about emotions as though they shared them – even when they don’t. In humans too, as already mentioned talking about emotions may cognitively distort underlying processes based on what people think their emotions should be, rather than accurately report emotional content (Wilson and Schooler, 1991; Dijksterhuis et al., 2006; Schooler and Mauss, 2010). In short, talking about emotions is often not a reliable window or ‘gold standard’ source of evidence into actual underlying emotions, even when verbal declarations of emotion are available.

## Applications of Animal Studies to Human Emotions

In animals as well as humans, psychologists can objectively map the shape of emotional/motivational processes from the outside, by finding the lawful rules that govern their operation in action, even when we don’t know what is subjectively felt on the inside (Nagel, 1974). This aims for a schematic understanding of other minds, rather than an introspective mirroring of phenomenal experience. Schematic understanding is the way we study perception, learning, memory and cognition in animals. It’s even how we measure and understand perception in people whose visual world differs from ours (e.g., detecting color blindness). What is a schematic understanding of emotion? As the comparative psychologists William Mason and John Capitanio put it, “*Our approach to the ontogeny of basic emotions is based on the phenomenon of component schemas. Schemas are hypothetical information-processing units, closely linked to observed behaviors. Most if not all schemas are affectively charged*” (Mason and Capitanio, 2012, p. 240). Similarly, the psychologists of emotion Nico Frijda and W. Gerrod Parrott, posit that animals as well as humans can express ‘ur-emotions,’ as objectively detectable psychological processes that combine appraisals, affective attitudes and action readiness. Their “ur” label is meant to imply that such emotional reactions may be relatively primitive and fundamental, which overlap but are not necessarily identical with traditional “basic emotions” categories (e.g., anger, joy, etc.). They suggest: “*each animal species has at its disposal some innate primary actions that implement the functions we have assigned to the ur-emotions” (p. 412), including the “ur-emotion of desire in its “urest” or purest or most target-free form: as mere wanting*” (Frijda and Parrott, 2011, p. 413). Mere ‘wanting’ refers to incentive salience, discussed below. Other psychologists and neuroscientists have expressed similar views about fundamental affective reactions in animals and human infants (Darwin, 1872; Berridge, 2000; Keltner and Ekman, 2000; Steiner et al., 2001; Damasio, 2004; Panksepp, 2011; Anderson and Adolphs, 2014; Berridge and Kringelbach, 2015; Frijda, 2016).

**FIGURE 1:**
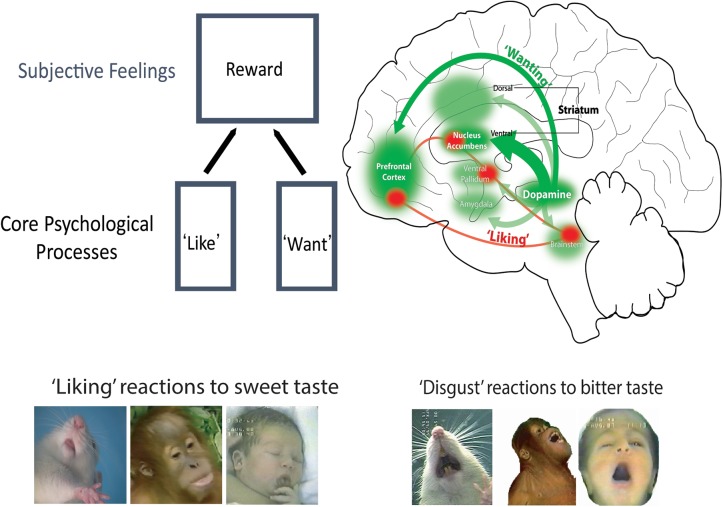
Brain systems of ‘wanting’ versus ‘liking’. Robust and large mesocorticolimbic circuitry can generate intense ‘wanting’ (green), including both mesolimbic dopamine projections and many of its target structures. By comparison, ‘liking’ is mediated by a smaller and relatively fragile set of ‘hedonic hotspots,’ which are distributed across the brain but act as an integrated network.

Of course, one goal of animal studies is to gain insights that have application to understanding human emotions and motivations. For that, we need to posit continuity between those affective mechanisms in animals and humans. Otherwise, it would be impossible to make the jump from discoveries about animal affective processes, to human affective processes or to human clinical disorders of addiction, depression, schizophrenia, etc. This jump is not always easy. Indeed another reason LeDoux gives for denying that animals are capable of fear is because animal fear conditioning studies failed to provide new effective medications for human anxiety or panic disorders. However, this lack of therapeutic development could just as well mean that Pavlovian fear learning (e.g., behavioral freezing to a sound that predicts footshock) was not the best animal ‘fear’ reaction for assessing the efficacy of fear-reducing medications. It is an unfounded leap to conclude instead it means that rats are incapable of fear. Other animal tests of fear or anxiety using different reactions and situations might be more successful. In any case, a counterargument has been made that many of the current anti-anxiety medications used by human patients actually were developed through various animal models of ‘fearful’ reactions (Treit et al., 2010; Bourin, 2015). And there are many other cases where animal studies of emotion have produced results with successful implications for understanding human psychology and disorders. I believe these include a few from my own lab, and one is the brain-based distinction between ‘wanting’ and ‘liking’ pleasant rewards (**Figure 1**), described next.

## ‘Wanting’ Versus ‘Liking’: Implications From Animals to Humans

The distinction between ‘wanting’ and ‘liking’ initially came as a surprise to me and to my colleagues. We had believed until the late 1980s that the two concepts necessarily went together, as two semantic sides of the same psychological coin. Evidence from our studies of brain mechanisms for food reward in rats forced us to change our minds. Today the distinction between ‘liking’ and ‘wanting’ now rests on 30 years of results in animal studies, and 15 years of confirmatory findings in humans. The distinction has also turned out to have applications to human affective disorders, ranging from addiction to schizophrenia, depression and Parkinson’s disease. But for the first decade of this hypothesis, the schematic understanding from animal evidence was all we had, based on objective affective reactions, which stood alone and pointed the way.

Our initial discovery came from studies of the role of brain mesolimbic dopamine systems in reward. In the 1980s, we and most other psychologists and neuroscientists believed that dopamine mediated pleasure or ‘liking’: the hedonic impact of reward. This was best expressed at the time by the dopamine pleasure hypothesis of the neuroscientist Roy Wise, who had suggested that mesolimbic dopamine signals are “*translated into the hedonic messages we experience as pleasure, euphoria, and ‘yumminess*”’ (Wise, 1980, p. 94). Conversely, Wise suggested an opposite anhedonia hypothesis for the psychological effect of drugs that blocked dopamine receptors (often called neuroleptic drugs or dopamine antagonist drugs), which posited those drugs to cause tasty foods and all other reward to lose their pleasure or hedonic impact. His evidence was that such drugs produced ‘extinction mimicry’: making animals or people gradually cease to pursue or consume the reward, as though their pleasure had been drained, similar to real extinction procedures in which expected reward are suddenly missing, causing animals to extinguish or gradually cease working on tasks that formerly produced the reward. To give credit where due, some other neuroscientists, such as John Salamone and Jaak Panksepp had also expressed early doubts about the dopamine-as-pleasure hypothesis. Salamone had disputed Wise’s anhedonia hypothesis around 1990, on the grounds that neuroleptic extinction mimicry did not fully mimic all features of real extinction, and because he noted that mild-to-moderate dopamine suppressions often reduced effort exerted for reward without necessarily also reducing reward consumption (Salamone, 1991; Salamone et al., 2015). Panksepp suggested what he called an ‘expectancy hypothesis’ for dopamine, in which it caused the “*heightened energization of animals searching for and expecting reward*” (while also contributing to positive affect in his view, as a combined motivational/hedonic incentive mechanism) (Panksepp, 1986, p. 91).

But I must admit myself to having been mostly convinced by Wise’s elegant evidence for mesolimbic dopamine as pleasure until we began to probe dopamine ourselves in a schematic approach to specifically measuring ‘liking.’ Dopamine then failed to live up to its hedonic reputation. To my initial surprise and disappointment, our results on sweetness ‘liking’ reactions in rats indicated that dopamine did not mediate pleasure ‘liking’ after all. We were using an objective measure of affective ‘yumminess’ in rats similar to that used by human parents for thousands of years to ask their newborn infants if they ‘liked’ the foods eaten by the families: affective facial expressions of ‘liking’ elicited by a small taste of the foods (Steiner et al., 2001). In human infants, sweet tastes typically elicit relaxed and rhythmic mouth and tongue movements, and licking of the lips. Bitter tastes oppositely elicit ‘disgust’ reactions of mouth gapes, headshakes, arm flails and so on. Some of these affective facial expressions to taste are shared by apes and other primates, and even by rats and other non-primates (Grill and Norgren, 1978; Berridge, 2000). Using this schematic measure of ‘liking’ versus ‘disgust,’ we found that drug disruption of dopamine failed to reduce facial ‘liking’ reactions to sweetness, contrary to our anhedonia-based prediction (Treit and Berridge, 1990). Further, even complete removal of nearly all dopamine by neurochemical brain lesions left rats’ sweetness ‘liking’ completely normal and unimpaired, despite eliminating all ‘wanting’ to eat (Berridge et al., 1989). Conversely, we found that stimulating mesolimbic systems with an electrode to promote dopamine release turned on intense ‘wanting’ to eat, but failed to increase ‘liking’ at all (Berridge and Valenstein, 1991). Later studies in our lab went on to identify various psychological signature features of dopamine-mediated ‘wanting’ or incentive salience, which will be described later (Wyvell and Berridge, 2000; Peciña et al., 2003; Smith et al., 2011; Pecina and Berridge, 2013). In short, the early animal evidence showed that ‘liking’ was different from ‘wanting,’ and that dopamine mediated ‘wanting’ but not ‘liking’ (**Figure 1**). But only animal evidence existed for these hypotheses until nearly 2000. That made ours a lonely position, because most affective neuroscientists still wrote of dopamine as a pleasure mechanism throughout the 1990s and often into the 2000s.

However, gradually a number of human studies began to emerge around 2000 that confirmed our dopamine-based liking/wanting distinction also applied to people’s feelings of pleasure produced by cocaine, heroin, and food reward. Studies began to report that blockade of human dopamine receptors by antagonist drugs, or dietary-induced reductions of dopamine release, did not reduce people’s subjective liking ratings for food pleasure, or for drug pleasures of cocaine or heroin (Brauer and De Wit, 1997; Leyton, 2010; Sienkiewicz-Jarosz et al., 2013). However, those dopamine suppressions did reduce people’s subjective reports of wanting to consume more of the drug or food reward.

Similarly, it is now increasingly clear that increases of human dopamine do not reliably cause enhancement of subjective ratings of pleasure (although many rewards do induce dopamine release as a consequence and correlate, but not the cause, of pleasure) (Leyton et al., 2002; Evans et al., 2006; Liggins et al., 2012). For example, giving ordinary people the medication L-DOPA, which produces surges in brain dopamine levels, does not increase their subjective ratings of pleasure feelings (Liggins et al., 2012). Dopamine surges in nucleus accumbens evoked by addictive amphetamine or by L-DOPA correlate poorly with human subjective liking ratings of the drug - but do control their subjective wanting ratings to take more of that drug (Leyton et al., 2002; Evans et al., 2006).

Thus, human confirmation began finally to amass for the ‘liking’ versus ‘wanting’ distinction we originally found in rats a decade earlier. Was our animal-based conclusion about human liking and wanting simply a lucky guess? We must have been very lucky indeed 10 years before the human confirmation began to emerge – unless we were accurately tapping in all along to ‘liking’ versus ‘wanting’ processes shared by animals and humans, which made it not a guess at all. Instead it was a schematic inference about hedonic and motivation mechanisms of reward gained from animals, with direct applications to human psychology and clinical affective disorders.

## Clinical Applications of ‘Liking’ Versus ‘Wanting’

The first clinical application was to drug addiction, based on the dopamine-as-‘wanting’ idea combined with my Michigan colleague Terry Robinson’s findings that addictive drugs – when taken repeatedly in binge-like fashion by susceptible individuals – induce long-lasting hyper-reactivity in mesolimbic dopamine-related systems, known as neural sensitization (Robinson and Berridge, 1993; Berridge and Robinson, 2016). Sensitized mesolimbic dopamine neurons release more dopamine when a drug is taken, their dopamine-receiving target neurons become more receptive to excitatory glutamate signals, etc. This neural sensitization creates dopamine hyper-reactivity to drugs and their cues in sensitized individuals. Neural sensitization can happen in many of the same brain dopamine-related neurons that undergo drug tolerance because the two changes proceed through parallel chains of molecular events inside neurons, almost like ships passing in the night. In the short run, tolerance and withdrawal often win and mask sensitization – as long as drugs continue to be taken. But unlike tolerance and withdrawal, neural sensitization doesn’t go away when the individual stops taking drugs. Then sensitization wins. That is because neural sensitization grows or ‘incubates’ over weeks of drug abstinence, so that craving becomes stronger, and once sensitization emerges it can last years in animals and humans (Paulson et al., 1991; Boileau et al., 2006; Li et al., 2015).

The implication for human addiction is that sensitized drug addicts could have intense cue-triggered ‘wanting’ for their drugs, especially when encountering drug cues in emotionally aroused states, even if their ‘liking’ for drugs declined (Robinson and Berridge, 1993; Berridge and Robinson, 2016). Originally we applied this incentive-sensitization theory only to drug addiction, because only drugs were then known to induce mesolimbic neural sensitization. However, it has subsequently become clear that it is also possible to induce mesolimbic sensitization without drugs in susceptible individuals, such as by exposures to traumatic stresses, exposures to strong specific appetites, etc. Recently it has even become plausible that some people, who may be especially vulnerable to developing mesolimbic sensitization, may become ‘spontaneously’ sensitized in particular situations via endogenous mechanisms, resulting in behavioral addictions.

There are strong individual differences in sensitization vulnerability, due to genes, hormones, previous experiences, etc. Presumably those who develop behavioral addictions are the most vulnerable individuals, able to develop mesolimbic sensitization via endogenous mechanisms, without need of drugs. Evidence that sensitization happens in behavioral addictions is that fMRI studies have reported individuals with gambling addiction, shopping addiction, internet addiction or sex addiction, to exhibit brain signatures of mesolimbic sensitization to appropriate stimuli: that is, neural hyper-reactivity to their own addictive cues, higher than non-addicted people show to the same reward, and higher than the same addicted individuals show to cues for other (non-addicted) rewards (Davis and Carter, 2009; Gearhardt et al., 2011; O’Sullivan et al., 2011; Hartston, 2012; Linnet et al., 2012; Ray et al., 2012; Voon et al., 2014). This sensitized brain reaction evoked by addictive cues should produce stronger cue-triggered psychological surges of ‘wanting’ than faced by other non-sensitized individuals. Those addictive urges would be further amplified when the cues were encountered during stress, emotional excitement, intoxication or other states that prime the reactivity of mesolimbic systems. This could produce extra-strong ‘wants’ in behavioral addictions that sometimes surprise even the addict by their intensity.

A recent inadvertent ‘medical experiment’ involving new medications for patients with Parkinson’s disease has provided further proof of principle for incentive-sensitization of ‘wanting’ in humans. New dopamine-stimulating medications have produced addictive-like motivations in people who were previously least likely to ever become addicts. This is called ‘dopamine dysregulation syndrome’ or DDS, and happens in 15% or more of Parkinson’s patients who are given newer ‘direct agonist’ medications that directly stimulate their brain dopamine D2/D3 receptors, especially at high doses. These drugs act as artificial dopamine on those receptors, skipping over the need for natural dopamine (older L-Dopa medication promoted natural dopamine synthesis, but did not directly stimulate receptors) (O’Sullivan et al., 2009). Receptor-stimulated DDS patients can become sensitized, and are reported to compulsively pursue incentive activities in an addictive-like fashion: gambling, shopping, sex, internet, hobbies, taking drugs or even over-consuming their medications in much higher quantities than intended by their physicians (Ondo and Lai, 2008; Callesen et al., 2013; Friedman and Chang, 2013; Politis et al., 2013). Many patients who pursue one of those activities may also pursue a second or a third, as their sensitized ‘wanting’ spills over into several potential targets and typically the compulsive motivations decline when the medications are reduced or stopped. Patients who show DDS compulsions have been suggested to also have neural features of incentive-sensitization, for example releasing more dopamine in nucleus accumbens than other patients when stimulated with L-Dopa. Compulsive motivations of DDS do not seem to involve higher pleasure ‘liking’ for the pursued reward once obtained, but rather exist alone as intense and often disturbing ‘wants.’ DDS symptoms are perhaps the most striking human confirmation of the hypothesis that mesolimbic dopamine activation can cause addictive ‘wanting,’ which originated from animal studies.

Beyond addictions, the liking/wanting distinction also has been applied to nearly the opposite psychological condition: namely, a motivational component of ‘anhedonia’ syndromes in schizophrenia, depression, and unmedicated Parkinson’s disease. These conditions have traditionally have been described as involving anhedonia or incapacity to experience pleasure (similar to the original Wise hypothesis that dopamine blockade reduced pleasure). However, recent studies suggest that many patients with schizophrenia or Parkinson’s, and possibly some with major depression, are not actually anhedonic after all: they report normal sensory pleasure ratings of ice cream or other sensory reward in the moment, even though they attach little value to any sensory, social or other pleasant reward in life. Some investigators therefore have suggested their deficit really reflects a selective loss of incentive motivation or ‘wanting,’ rather than loss of pleasure ‘liking’ (Sienkiewicz-Jarosz et al., 2005; Dowd and Barch, 2010; Treadway and Zald, 2011; Barch et al., 2014). They call this motivational deficit ‘avolition,’ ‘anticipatory anhedonia’ or ‘motivational anhedonia,’ to contrast it with true ‘anhedonia’ or ‘consummatory anhedonia’ that would reflect loss of pleasure *per se*. Like addiction, these clinical applications draw on a psychological understanding of the separability of ‘wanting’ from ’liking’ that originally came from animal studies.

### History of Motivation Concepts in Psychology and Neuroscience

Now we will shift gears, and turn to a very different question. Namely, the nature of motivation processes themselves, and how motivations actually control behavior. Motivation concepts have changed a lot over the last few decades. For example, from 1900 to the 1970s, much of the thinking about reward motivation in psychology and neuroscience was dominated by two concepts: drive and drive reduction (Hull, 1951; Miller, 1971). Drive was typically conceived as an aversive state that goaded behavior into action to reduce the unpleasant drive (hunger, thirst, sex, drug withdrawal, etc.). We’ll focus on here natural hunger and thirst, and on drug addiction, as clearest examples.

Drive reduction served as reward in this motivational framework; not as a positive pleasure or incentive, but in a negative reinforcement sense of eliminating the aversive drive. The notion that motivation is mostly to escape *unpleasant* states is a bit reminiscent of William James’ quip in a 1901 letter on happiness (published with other letters in 1920) written shortly after his exhausting experience of delivering the famous Edinburgh Lectures that he subsequently revised into a book (James, 1902). “*Happiness, I have lately discovered, is no positive feeling, but a negative condition of freedom from a number of restrictive sensations of which our organism usually seems the seat… When they are wiped out, the clearness and cleanness of the contrast is happiness. This is why anesthetics make us so happy. But don’t you take to drink on that account*” (James, 1920, p. 158).

Natural motivations were viewed by drive-reduction theory as generators of aversive states, triggered by signals for physiological homeostatic needs (low nutrient reserves for hunger, dehydration for thirst) or of other forms of deprivation (lack of sex, withdrawal from addictive drugs, etc.) (Hull, 1951; Miller, 1971). Their respective rewards were all viewed to reduce the unpleasant drives, by consuming the drive’s goal object until the aversive deficit state was gone (food, water, copulation, addictive drug, etc.). Today, drive reduction theories can still be found in a few cases, such as theories of drug addiction based on withdrawal symptoms, or in the conclusions of a few recent neuroscience studies of hunger or thirst.

For example, in drug addiction, the opponent-process theory posited negative hedonic states of withdrawal and dysphoria to be the chief force driving addicts to take drugs (Solomon and Corbit, 1974), a view that still has adherents (Koob and Volkow, 2016; Keramati et al., 2017). Modern proponents suggest that downregulation or loss of brain dopamine D2 receptors (one of the two main types of neuronal receptors for dopamine) make addicts experience less pleasure in their lives than other people (Koob and Volkow, 2016; Keramati et al., 2017). Consequently, these theorists argue that addicts must take addictive drugs to achieve normal levels of dopamine stimulation and pleasure. However, this hedonic deficiency view has been critiqued on grounds that dopamine receptor downregulation is probably a consequence of drug taking rather than the original cause of addiction, on grounds that dopamine does not actually cause pleasure nor does dopamine downregulation cause pleasure deficits, and on grounds that addictions persist after withdrawal syndromes go away (Berridge and Robinson, 2016).

Recent studies of hunger and thirst neuronal circuitry provide a few more examples that drive reduction ideas persist today. Hunger neurons are thought to include those in the hypothalamus that release agouti-related peptide (AgRP), and stimulating those neurons cause mice to eat. However, a mouse may subsequently avoid a flavor that was paired with the AgRP neuronal stimulation that made it eat, leading some authors to suggest that stimulated hunger is necessarily an aversive drive that the mouse later associates with the flavor (Betley et al., 2015). However, other neuroscientists have found that mice will actually work to turn on their hypothalamic AgRP neurons that previously made them eat, indicating that the increased appetite can be dissociated from negative valence, and even take on a positive valence in the right conditions (Chen et al., 2016). This makes a pure drive reduction theory of AgRP hunger less plausible, similar as to what happened for hypothalamic reward electrodes discussed below. Recently, a thirst drive-reduction hypothesis was suggested by authors of a study similar to the first AgRP one for hunger (Allen et al., 2017). These thirst-study authors found that artificial stimulation of neurons in the hypothalamus made mice drink, but that mice would work to turn off the stimulation if given a choice, thus leading the authors to suggest that thirst is essentially aversive.

Yet if history repeats itself, the aversiveness of the hypothalamic neuronal activation may in future turn out to be separable from its ability to trigger drinking (Toates, 1986; Berridge, 2004). If so, aversiveness above could in part due to relatively extreme or unnatural parameters of the earlier neural stimulation. The crucial test will be if future studies with other stimulation parameters can eventually tease apart drinking elicitation from aversive effects – possibly even finding that activation of the drinking neurons can become sought under some circumstances. That could help dissolve these new drive-reduction explanations into a future incentive motivation explanation for the same hypothalamic circuitry. This doesn’t deny that intense hunger, thirst, etc., can be unpleasant. Of course they can, giving drive-reduction theories an eternally intuitive appeal. However, the brain’s motivation circuitry may be mostly oriented toward incentive processes, even for hunger, thirst, and the other biological motivations that drive reduction theory originally was invented to explain.

### The Fall of Drive Theories

Perhaps the single most compelling piece of evidence that moved motivation theory away from negative drive theories to positive incentive theories of motivation came from 1960s studies of reward and hunger motivation caused by brain electrode stimulation – mostly at sites in the lateral hypothalamus that indirectly also activated mesolimbic dopamine systems (Olds and Milner, 1954; Valenstein et al., 1970; Miller, 1971). It quickly became clear that brain reward electrodes were not only rewarding (in the sense that rats and people worked eagerly to activate them), but the same electrodes were also often powerfully motivating in the sense of apparently turning on natural motivations. The electrodes may actually not have caused much pleasure ‘liking’ after all, but they certainly activated ‘wanting’ for a variety of natural incentives (Berridge and Kringelbach, 2015). That is, simply giving free stimulations of the electrodes, without making them work for it, caused rats to suddenly start eating, or start drinking, or begin engaging in sex, or parental behaviors, etc. (Valenstein et al., 1970). Similarly in human psychiatric patients who had been implanted with similar brain electrodes, activation of a reward electrode caused sudden sexual urges, or urges to drink, or other intense motivations, in addition to supporting button pressing to self-stimulate their electrodes (Moan and Heath, 1972).

The finding that the same electrode was both rewarding (in the sense of being sought after) and motivating confounded expectations based on drive theories. According to drive theories, the intense motivations had to be aversive. According to drive reduction theories, reward was produced by reducing motivations, not by increasing drives. Thus behavioral neuroscientists of the time expected that an electrode that increased sex or hunger or thirst motivations, would be a punishing electrode. They expected that a reward electrode would reduce drive, and so stop any ongoing eating, drinking, or sex behavior – never stimulate those motivations. To drive theorists, a reward electrode that also increased motivation is an inexplicable paradox (Miller, 1973; Olds, 1973; Stuber and Wise, 2016).

How can that paradox be resolved? The fact that an electrode that caused eating behavior (or drinking, or sex, etc.) was also a reward electrode meant that the reward could not be due to drive reduction. Since the rewarding electrode actually increased the apparent drive to eat, reward needed to be understood as a phenomenon that was independent from drive reduction. The paradox was eventually solved by incentive motivation theory described below.

Other evidence has indicated similarly that the aversiveness of drives does not actually motivate much behavior, even for hunger and thirst. Nor is the reduction of an aversive drive actually the chief target of those motivations. For example, when in a place that has been repeatedly associated with hunger, or paired with thirst, one might expect a re-encounter of that place to trigger Pavlovian conditioned (cue-triggered) hunger or thirst again, and increase eating or drinking. That should happen if re-activation of the aversive need-state or drive motivates eating or drinking behavior. But that does not happen. For example, rats typically fail to increase eating in a place previously paired with hunger drive, nor to increase drinking in a place paired with thirst drive (Huston, 1972; Mineka et al., 1972; Toates, 1986). Pavlovian cues for hunger fail to elicit ingestive behaviors, that is, unless those cues were additionally paired with the *opportunity to eat* while hungry, or the *opportunity to drink* while thirsty. However, if that extra pairing with food consumption while hungry is given, then later the Pavlovian food cue can evoke conditioned eating even if encountered when the rat is no longer hungry (Weingarten, 1983; Holland et al., 2002; Petrovich, 2011). That is, a food incentive cue elicits eating, but a hunger cue does not. Similarly, for thirst,: encountering a place paired with thirst drive does not alter behavior, but encountering a *cue* for *water* will evoke intense increases in activity in thirsty rats, as though searching for water (Campbell, 1960). In these cases, cues for the *incentive* stimulus evoke motivation in a state, whereas cues for the *drive* state by itself do not. Hunger acts to enhance the incentive value of food, and thirst enhances the incentive value of water – but in the absence of incentives or their learned expectation, neither need state effectively motivates behavior as posited by aversive drive theory.

Not only is drive a weak motivator by itself, but drive reduction turns out to be surprisingly impotent as a reward by itself. Reduction of a hunger or thirst drive is usually not enough to reinforce behavior, unless an incentive stimulus is also involved. For example, rats that can deliver nutrients directly to their stomach via feeding through a gastric fistula in some studies fail to bar press for the nutrients at all (Holman, 1969). However, if opportunity to taste a mouthful of saccharin at the same time as a gastric nutrient infusion, then the rats do learn to bar press for the combination (the sweet taste by itself is similarly insufficient, revealing an importance of interaction between incentive stimulus and physiological state, captured by Bindra-Toates rules of incentive motivation described below) (Holman, 1969). Even studies that succeeded in getting rats to work for intra-gastric delivery of nutrients reported only weak effects: such rats bar press to earn only about 30% of their normal intake of calories (Nicolaidis and Rowland, 1975). Similarly, hungry rats merely walked slowly to a place where they expect intra-gastric milk, but eagerly ran to a place where they could drink and taste the milk while hungry (Miller and Kessen, 1952). And drive reduction is not even satiating: reducing a physiological drive often does not effectively reduce the motivated behavior. For example, delivering daily calories intravenously to dogs, who were also allowed to eat actual food normally if they wished, did not suppress their daily eating intake: the dogs continued to eat customary amounts of food in addition to their full doses of intravenous nutrients, and so soon became obese (Turner et al., 1975). All these observations indicate that the role of physiological ‘drive states’ is really to amplify the incentive value of their relevant incentive targets, and magnify their reward properties, rather than act as independent drives (Toates, 1986; Berridge, 2012).

## Incentive Motivation: Relating ‘Liking’ to ‘Wanting’

What is the alternative to drive theories? Incentive motivation theories posit that motivation is directed toward affectively positive incentives, and that brain motivation systems modulate those incentive values (Toates, 1986; Berridge, 2004). Hunger, thirst, and other motivation states primarily act to *enhance* the incentive value of their particular reward, increasing ‘wanting’ and ‘liking’ for foods, or for water, and so on, rather than acting primarily as aversive goads (Toates, 1986; Cabanac, 1992; Dickinson and Balleine, 2002).

Incentive motivation is focused on reward, which involve three categories of mechanism: wanting, liking and learning. ‘Liking’ reactions reflect the hedonic impact of pleasant reward, and is often considered the essentional kernal of reward. What in the brain is responsible for ‘liking’ reactions? Our research findings have indicated that pleasure ‘liking’ is generated by an anatomically small, neurochemically restricted, and functionally fragile brain circuit – which leaves out the large dopamine projection system of ‘wanting’ (Berridge and Kringelbach, 2015). The relative fragility of ‘liking’ circuitry, compared to the robustness of ‘wanting’ circuitry, may be one reason why intense pleasures are fewer and farther between in life than intense desires. Only a network of small ‘hedonic hotspots’ is able to amplify ‘liking’ reactions to a sensory pleasure, such as sweetness.

**FIGURE 2:**
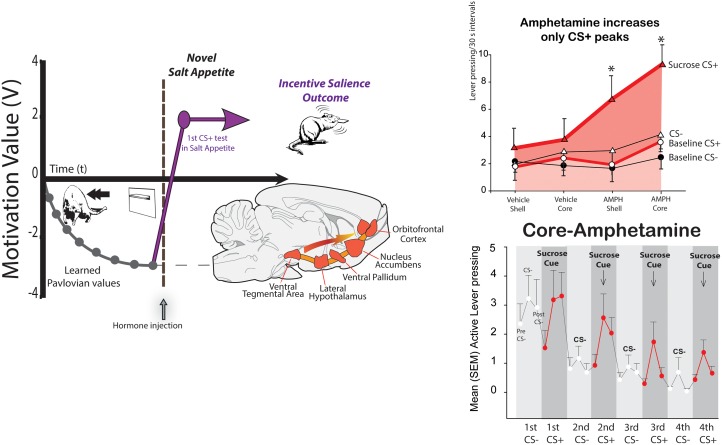
Synergy of cues and internal state in generating intense ‘wanting.’ A Pavlovian cue lever for disgusting Dead Sea saltiness becomes negatively repulsive during learning **(left)**. But in a novel salt-appetite state, the cue becomes immediately ‘wanted’ and attractive, by activating mesocorticolimbic circuitry for incentive salience (Robinson and Berridge, 2013). In Pavlovian-Instrumental transfer (PIT; right), a Pavlovian reward CS+ cue (sound) elicits small peaks of ‘wanting’ during extinction test **(bottom)**. An amphetamine microinjection that releases dopamine in nucleus accumbens selectively magnifies only the CS triggered peaks of cue-triggered ‘wanting,’ without raising baseline effort based on act-outcome expectations or the effect of an irrelevant CS- control sound (Pecina and Berridge, 2013).

**FIGURE 3:**
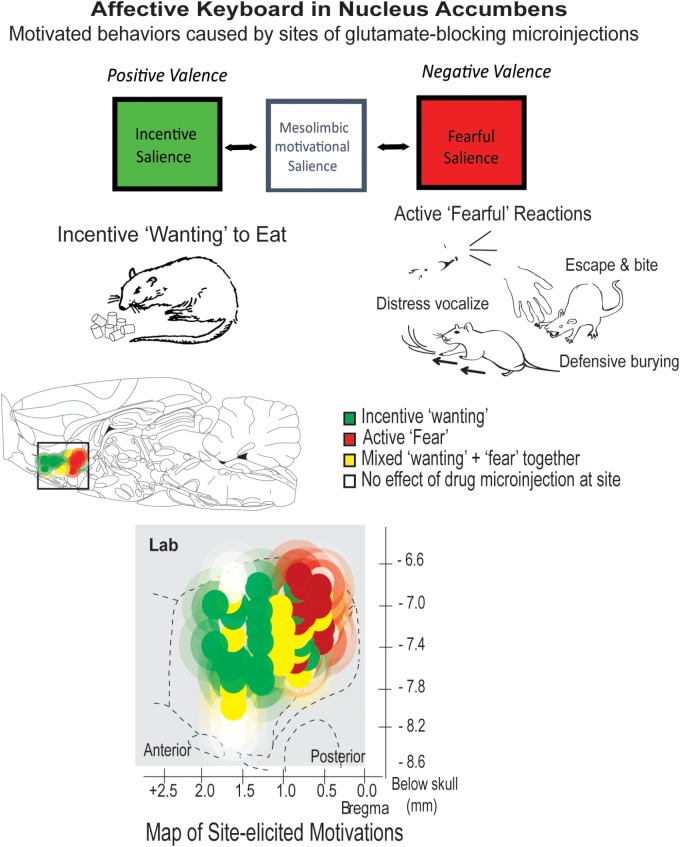
Motivational salience: positive ‘wanting’ versus negative ‘fear.’ The nucleus accumbens can generate either incentive salience or fearful salience. Microinjections of the same glutamate-blocking drug (DNQX) elicit opposite motivations at different sites. Both incentive motivation and fearful motivation require local mesolimbic dopamine. Many individual sites can be flipped back and forth between generating ‘wanting’ and ‘fear’ by changes in environmental ambience, as described in text. Based on Reynolds and Berridge (2008) and Richard and Berridge (2011).

Hedonic hotspots are anatomically small pleasure-generating islands of brain tissue, tucked within larger limbic structures, such as nucleus accumbens and limbic cortex (**Figures 2**, **3**). The size of each hotspot discovered so far is only a cubic millimeter or so in volume in the brain of a rat. In the brain of a person, a hotspot would be expected to be about a cubic centimeter in volume, extrapolating from the size difference between whole brains.

Neurochemically, these hotspots respond with hedonic amplification to opioid, endocannabinoid, orexin and related neurotransmitter signals – but never dopamine. When stimulated with those neurochemical microinjections, the hotspots increase ‘liking’ reactions to double or triple normal levels. But no ‘liking’ enhancement occurs if the same drug microinjections are moved outside the boundaries of the hedonic hotspots, even still within the same structure (e.g., nucleus accumbens). Instead the microinjection at those sites outside the hotspot (or dopamine within the hotspot) still will stimulate only intense ‘wanting’ in the remainder of the structures, without enhanced ‘liking’ and sometimes even while suppressing ‘liking.’

Several of these hedonic hotspots have been found in the brain scattered from cortex to brainstem (**Figure 1**). Hotspots are found in limbic areas of prefrontal cortex such as in orbitofrontal cortex, in insula cortex, and in subcortical structures such as nucleus accumbens, ventral pallidum (the chief target of nucleus accumbens), and the brainstem pons. The distributed hotspots appear to act together as a functionally interconnected network, so that stimulating one hotspot with a drug microinjection causes other hotspots to be recruited into neurobiological activation too (Smith and Berridge, 2007; Castro and Berridge, 2017). Unanimous activation of multiple hotspots together appears required in order to amplify sensory pleasures. This is shown by observations that if unanimity is prevented (by simultaneously suppressing one hotspot while stimulating another) the ‘liking’ enhancement ordinarily produced by the stimulation will be prevented. However, a ‘wanting’ increase may still may occur in such a case, again illustrating the more robust nature of ‘wanting’ circuitry (Smith and Berridge, 2007; Berridge and Kringelbach, 2015).

### Psychological Features of Incentive Salience as ‘Wanting’

Incentive salience, is a mesocorticolimbic form of wanting, sometimes called ‘wanting’ (with quotation marks) to distinguish it from cognitive forms of wanting. Cognitive wanting is goal-oriented, and based typically on declarative memories and on cognitive expectations of act-outcome relations, and less tied to mesolimbic dopamine-related systems. By contrast, incentive salience has distinct signature features: often being cue-triggered as a temporary peak of desire to obtain or consume an associated reward (Berridge, 2001, 2012). Incentive salience makes those reward cues attractive and attention-grabbing, eliciting approach and giving them a ‘motivational magnet’ property (Saunders and Robinson, 2013; Flagel and Robinson, 2017). Sometimes the cues may even become targets of consumption themselves. Incentive salience is responsible for many incentive motivation phenomena described above, including the apparent paradox that the same brain stimulation can be both motivating (i.e., stimulate eating, drinking, etc.) and rewarding (i.e., sought after in self-stimulation tests) at the same time.

Normally, mesolimbic ‘wanting’ and cognitive wanting often go together. Incentive salience ‘wanting’ gives a motivationally compelling quality to a cognitive desire, and helps motivate action to obtain the goal. Incentive salience is often thus a spur to action, reflecting the overlap between dopamine functions of motivation and of movement (Salamone et al., 2015, 2018). Thus incentive salience may proactively facilitate action and engagement (Ryan and Deci, 2000; Carver, 2009; Kruglanski et al., 2014). In people, also, strength of mesolimbic incentive salience has also been linked to personality traits of impulsiveness and sensation-seeking (e.g., Gray’s BAS [behavioral activation system]) (Gray et al., 1999; Beaver et al., 2008; Hickey and Peelen, 2015).

Incentive salience mechanisms look for potential targets and triggers in the world, either in physical stimuli or in imagination. Many reward in life are learned, so that their ‘wanting’ is activated by associated cues, but interaction with world targets can also be seen in people who suddenly have their mesolimbic dopamine systems artificially activated for the first time, such as by a newly-implanted brain stimulation electrode. For example, a woman whose subthalamic electrode (activating an input to dopamine systems) was turned on for the first time suddenly “*was excessively talkative and it was not possible to interrupt her while she was speaking,”* and subsequently with further stimulation “*was in love with two neurologists, and tried to embrace and kiss people*” (p. 1383) (at least, in love according to the neurologists who published the report) (Herzog et al., 2003). Similarly, a man in Germany whose nucleus accumbens electrode was first turned on, “*spontaneously reported that he realized that he was in the (city of) Cologne, that he never visited the famous Cologne Cathedral, and he planned on doing this in the immediate future, which he indeed did the day following the operation*” (p. 372) (Schlaepfer et al., 2008). The flexibility of such suddenly enabled motivation in seeking targets overlaps with psychological concepts of general motivational engagement (Higgins, 2006). However, with repeated activation, learning mechanisms tend to focus amplified incentive salience more narrowly on to a particular ‘wanted’ target, with addiction being an extreme example of a too-narrowly focused ‘want.’

### Theoretical Roots

Incentive salience follows psychological rules described originally by a useful incentive motivation framework posited successively by the psychologists Dalbir Bindra and Frederick Toates (Bindra, 1978; Toates, 1986). Dalbir Bindra posited in 1970s that Pavlovian incentive cues or conditioned stimuli (CS), which predict pleasant reward as unconditioned stimuli (UCS), take on some of the motivation features of their associated UCS reward (food, drink, sex, drugs, etc.). These cue features include the ability of CSs to trigger motivated ‘wanting’ to seek and consume their reward, and to a degree also trigger hedonic ‘liking.’

Frederick Toates suggested in the 1980s that reward cues additionally participate in a CS-state interaction, similar to a reward UCS-state interaction called alliesthesia. Alliesthesia refers to the phenomenon that food becomes more hedonically rewarding when we are in a state of hunger, water is more rewarding when thirsty, nasty seawater becomes a rewarding taste if we are sodium-deprived, warmth is more rewarding when we are cold, coolness more rewarding when we are hot, and so on (Cabanac, 1971). Toates suggested that a learned cue or CS similarly interacted with an individual’s current internal state to generate incentive motivation. That is, an incentive percept (food or associated cues) would generate intense motivation when perceived in a physiologically hungry state, but not so much when perceived in a satiated state.

This interaction can be very powerful, able even to reverse the affective valence of a learned cue. For example, is it possible to suddenly intensely ‘want’ something that you’ve learned is always disgusting? Yes, says incentive salience theory, because of this interaction.

Take a rat that has learned that a lever CS suddenly appearing through a slot in the wall, predicts a mouthful of Dead Sea seawater (three times saltier than ocean seawater; delivered painlessly through oral cannulae that had been surgically implanted weeks before) (Robinson and Berridge, 2013). The intense saltiness is disgusting, and elicits mouth gapes, headshakes, arm flails, etc. The rat will shrink from the predictive CS lever and try to escape, whenever it appears. But what if the rat wakes up on one day in a new salt appetite state of sodium depletion that it never before experienced. Squirrels and deer in the woods are familiar with salt appetite, as were our human ancestors, but modern people and modern lab rats have more than enough NaCl in our foods, so that we have never in our lives been sodium deficient. Yet salt appetite can be induced overnight in a rat by drugs that make the body quickly lose sodium, and which produce hormonal aldosterone and angiotensin II signals to its brain that mimic those of a natural salt appetite. Now its response to the metal CS is immediately changed, and the lever CS becomes suddenly ‘wanted,’ even though it has so far only tasted actual salt infusions as ‘disgusting’ (Robinson and Berridge, 2013). In the new state, the rat will jump on the formerly-repulsive lever as soon as it appears, and nibble and lick the metal lever as avidly as if it predicted sugar water (**Figure 2**). The transformation of the CS-triggered motivation needs no new learning – it happens even before the rat ever re-tastes Dead sea saltiness in its newly ‘liked’ status. How does this happen? At the moment the rat re-encounters the salt-cue lever in newly depleted state, its mesocorticolimbic dopamine brain system of incentive salience is triggered into high activity, evident neurally as dopamine-related neurons start transcribing genes such as c-fos into protein such as Fos, to trigger metabolic activation of neuronal functions. The cue/state encounter triggers these Fos increases in neurons of ventral tegmentum (location of dopamine neurons), nucleus accumbens (chief target of mesolimbic dopamine projections), and ventral pallidum (the next target of nucleus accumbens outputs), and in limbic prefrontal cortex regions (which also receive dopamine projections), such as orbitofrontal cortex and anterior cingulate cortex (Robinson and Berridge, 2013). This cue-state interaction allows the CS to elicit intense incentive salience.

In this way, Pavlovian cue-triggered incentive salience can sometimes be smarter than a cognitive system: a cognitive system needs to act upon past knowledge of goal values that it has gained from experience (Dayan and Berridge, 2014). A cognitively rational rat or person, would be guided by memories of previous disgusting saltiness, and would need to retaste the saltiness in newly ‘liked’ status, in order to update goal values as positive required to seek the salt or its cue (Dickinson and Balleine, 2010). Both people and rats can behave ‘rationally’ in new salt appetites – at least, if guiding salt-related cues are absent. This may be why people who experience a novel salt appetite sometimes report feeling slightly ill, but do not necessarily crave salt (Leshem, 2009). However, once a person encounters salt or its Pavlovian cues in the deficient state, incentive salience kicks in and the appetite can become intense and focused into a desire to consume handfuls of salt (Wilkins and Richter, 1940).

Such reversals in the incentive salience of cues is a powerful confirmation that mesocorticolimbic incentive salience mechanisms obey Bindra-Toatesian rules. In ordinary life, shifts in cue-triggered ‘wanting’ may be only incremental within a single valence, merely varying between zero and high. However, the motivational power of cues is modulated by a host of physiological factors, ranging from appetites and satieties, to drug intoxication, stress and emotional arousal states, etc. Reward cues alone are relatively powerless at producing motivation without an appropriate brain state. It’s the brain’s reaction to a cue that matters for ‘wanting’ motivation via incentive salience (Berridge, 2012; Dayan and Berridge, 2014).

But the cue-state interaction for incentive salience also works in the opposite direction, so that encounters with particular cues can vary the motivational power of relevant physiological states. That’s why mere drive or drive reduction is usually not enough to motivate. It also applies to drug-induced motivation. For example, an hour of continuously high levels of brain dopamine, produced in a rat after microinjection of amphetamine in nucleus accumbens, does not actually cause continuously high ‘wanting’ motivation. Instead, its intense peaks of ‘wanting’ to obtain a reward come and go during the session together as reward cues are presented and withdrawn (Wyvell and Berridge, 2000; Pecina and Berridge, 2013; **Figure 2**). The dopamine elevation makes the cues trigger much higher surges of ‘wanting’ than they ordinarily would, but these surges decay within a minute or so once the cue disappears, although the dopamine remains continuously high. Conversely, other drugs can reduce the motivating power of reward cues, without disrupting their ability to convey learned information (Cogan et al., 2018).

Humans don’t necessarily require physical reward cues – vivid imagery about the reward may be enough to trigger limbic brain activations of incentive salience in people that would require cues in animals (May et al., 2004; Kochel et al., 2011; Jauregui-Lobera et al., 2012; Miyapuram et al., 2012). Imagination lets humans manufacture our own vivid mental temptation-provoking cues. But actual cues are still potent triggers of ‘wanting’ even for people. For example, in the classic Walter Mischel experiments on self-control, children were offered the choice of one marshmallow immediately or two marshmallows later if they could wait a few minutes. Children found it much harder to wait if any marshmallows were actually present to be seen and perhaps smelled (Mischel et al., 1989). The physical stimulus of a marshmallow in the present, unavoidably triggering vivid thoughts of its taste and what it would feel like to eat it, is a potent temptation for a child, even more than its imagination in the future. Indeed, children who successfully waited often employed strategies such as turning away from the marshmallow, thinking of the visible marshmallow as merely a photograph, or distracting themselves by imagining instead the taste of salty pretzels to redirect ‘wanting’ toward that different food. Of course, adults, and especially addicts, are vulnerable to cue-triggered temptations too.

### Dopamine Sensitization of ‘Wanting’ in Addiction

Incentive salience synergy between a reward cue and a hyper-reactive mesocorticolimbic brain state can sometimes work against our interest. This is why it can be unwise to shop while hungry. And some ‘wanting’-enhancing states of hyper-reactivity are more long lasting, such as dopamine-related neural sensitization. Addiction is the most extreme example, as neural sensitization makes brain dopamine systems more permanently hyper-responsive to drugs and drug cues, triggering strong urges to relapse and actually take drugs. Sensitized hyper-reactivity can be further amplified to even higher levels at certain moments by states of stress (whether unpleasant or celebrational), emotional excitement – or by taking a hit of drug again – creating a special window of heightened vulnerability to relapse. Sensitized ‘wanting’ in addicts creates a probabilistic form of cue-triggered compulsion, in the form of exaggerated temptation that can come by surprise, be amplified further by stress, and be hard to resist (Berridge and Robinson, 2016).

### Counter-Intuitive Sharing Between ‘Wanting’ and ‘Fear’

Incentive salience may additionally have a surprising and affectively-opposite cousin, in the form of negatively-valenced fearful salience. That is, reward ‘wanting’ and an active form of ‘fear’ reaction share overlapping mesolimbic dopamine and nucleus accumbens mechanisms of motivational salience despite being psychological opposites (Reynolds and Berridge, 2008; Richard and Berridge, 2011). Psychologically, both forms of motivational salience make their external stimulus triggers become attention-riveting and motivationally meaningful (Higgins, 2006). But while incentive salience makes its stimulus target also attractive and sought-out, fearful salience makes its stimulus target be perceived as threatening, evoking active coping responses and sometimes even defensive attack. Neurobiologically, both forms of motivational salience involve mesolimbic dopamine projections, and dopamine signal interactions with neuronal mechanisms in nucleus accumbens. The active form of ‘fearful’ reaction produced by this nucleus accumbens dopamine interaction contrasts to the more passive freezing reactions elicited by a threatening Pavlovian cue best known to depend on amygdala circuitry (LeDoux, 1996).

The active ‘fearful’ reactions are revealed by manipulations of the nucleus accumbens in rats (Reynolds and Berridge, 2008; Richard and Berridge, 2011). The nucleus accumbens’ shell compartment contains a desire-dread valence keyboard, organized from front to back (**Figure 3**): the keyboard is demonstrated by inhibitory microinjections of drugs that are either glutamate AMPA antagonists or GABA agonists. Each microinjection can be imagined as tapping a valenced key. Tapping keys in the front of this structure elicits strong ‘wanting,’ reflected as large increases in eating (doubling or quadrupling food intake), or creating a desire to return to the place where the key was tapped. Tapping keys in the posterior nucleus accumbens shell with the same drug microinjection oppositely elicits strong active ‘fear’ reactions. For example, a rat after a posterior microinjection may respond to the sight of people in the room with anti-predator reactions that rodents ordinarily emit toward threats, such as when a mother ground squirrel kicks sand with her forepaws toward an rattlesnake that approaches her pups in a burrow (Coss and Owings, 1978). Further, if a person gently attempts to pick up a rat when in this accumbens-induced ‘fearful’ state, the rat may even bite the newly-offensive hand, or scramble frantically in attempt to escape, even if the same rat is normally tame and friendly at all other times (Reynolds and Berridge, 2008).

The valence of sites in the nucleus accumbens keyboard is not determined solely by neuroanatomy. Valence of many sites can be partly or wholly retuned by psychological factors such as external ambience (Reynolds and Berridge, 2008), reflecting appraisal processes similar to human social psychology (Schachter and Singer, 1962; Higgins, 2006). For example, a bright and noisy environment flips sites in the front-middle of nucleus accumbens that normally generate ‘wanting’ into instead generating ‘fear,’ reversing from positive valence to negative valence. Conversely, a soothing dark and quiet environment flips many otherwise negative ‘fear’-generating sites in the middle-posterior of nucleus accumbens into generating positive ‘wanting’ (Reynolds and Berridge, 2008). Only anatomical extremes resist retuning, so that far-anterior sites remain resolutely appetitive while far-posterior sites remain ‘fear-generating,’ regardless of external ambience (**Figure 3**).

Both the ‘wanting’ and the ‘fear’ generated by these brain manipulations require mesolimbic dopamine signals in the nucleus accumbens. Simultaneously blocking all dopamine receptors at the microinjection site is functionally equivalent to removing the glutamate-blocking drug contents from the microinjection: no intense motivations are generated at all (Richard and Berridge, 2011). However, positive versus negative valence generated by glutamate antagonist microinjections depend on slightly different types of dopamine neuronal receptors. Positive ‘wanting’ required only D1 types of receptor activation by dopamine, whereas negative ‘fear’ required activation of both D1 and D2 types, which implicates additional neuronal circuitry (Richard and Berridge, 2011).

In people, this ‘fearful salience’ may be responsible for drug-induced states of paranoia in psychostimulant users, such as when euphoria flips to paranoid fear or aggression after high doses of methamphetamine or cocaine. In schizophrenic patients, a similar negatively-valenced role of dopamine in nucleus accumbens has been suggested to produce motivational paranoia that can accompany delusions of persecution (Howes and Kapur, 2009). This hypothesis is based on evidence of mesolimbic dopamine hyperactivity in schizophrenia, and also on the ability of anti-dopamine drugs that block D2 receptors to treat schizophrenic symptoms. By this ‘fearful salience’ hypothesis, medication-induced dopamine blockade may quickly reduce the motivational salience or compelling quality of perceptions and delusions, thus reducing the paranoia and providing a psychological distancing that more gradually leads to dissipation of cognitive delusions (although dopamine dysfunction is probably not the mechanism of the delusions themselves) (Howes and Kapur, 2009).

### Adding ‘Disgust’ to ‘Fear’ in Nucleus Accumbens

Disgust seems quite a different psychological emotion from fear (Rozin et al., 2008; Chapman and Anderson, 2012). In animals, both ‘disgust’ and ‘fear’ reactions could be called negatively-valenced defensive reactions by LeDoux’s recent terminology based on evolved functions (LeDoux J.E., 2015). But the functional ‘defensive’ label does not capture their psychological and neural distinctiveness. This points again to the need for emotion-based labels for these core psychological processes, even in animals, such as ‘fear’ and ‘disgust’. ‘Fear’ defends against external bodily danger threats, while ‘disgust’ defends against oral and internal toxic threats. However, neural mechanisms that generate ‘disgust’ and ‘fear’ reactions are mostly distinct, though they do partly overlap in the posterior shell of nucleus accumbens (Faure et al., 2010; Maren et al., 2013; Ho and Berridge, 2014; Perusini and Fanselow, 2015; Paré and Quirk, 2017).

We’ve studied core ‘disgust’ reactions such as gapes and headshakes that are naturally elicited by bitter tastes in rats (Steiner et al., 2001). In humans, disgust and fear expressions have nearly opposite statistical patterns of facial configuration, giving rise to the suggestion that these facial expressions may oppositely modulate sensory feedback: fear expressions may enhance relevant sensory perception (e.g., eye widening), whereas disgust may suppress relevant sensory perception (Susskind et al., 2008). Original sensory taste disgust may have been extended in human evolution to include also conceptual forms of disgust, such as in perceived physical contaminations (e.g., by germs, mutilation or putrefaction), or moral contaminations (e.g., particular acts of violence or sex or prejudice) (Rozin et al., 2008; Chapman et al., 2009). Human neuroimaging studies of disgust have often implicated the insula in cortex and subcortical striatal circuitry (which can include the nucleus accumbens) (Calder et al., 2007; Chapman and Anderson, 2012). Our studies in rats have further revealed that the only brain site where small lesions are able to cause sweetness to become perceived as nasty, and to elicit excessive ‘disgust’ reactions, is the hedonic hotspot of ventral pallidum, a striatal target which receives dense nucleus accumbens projections (Ho and Berridge, 2014). In short, despite both being defensive and negatively valenced fear and disgust are quite different emotional reactions.

Yet our animal brain-manipulation studies show one interesting overlap in neural generators for intense ‘disgust’ and ‘fear’ reactions in nucleus accumbens, involving a shift in neural mode of function at the same brain location. The posterior portion of its medial shell has two ‘fear’ generating neurochemical modes (glutamate AMPA receptor blockade versus GABA receptor activation), and only the GABA mode also generates excessive ‘disgust’. In this GABA-related mode, the normally ‘liked’ taste of sucrose instead elicits ‘disgust’ gapes and headshakes from rats, as though the taste were bitter quinine. This ‘disgust-plus-fear’ mode is produced when the posterior shell zone receives microinjections of a drug that activates GABA receptors, such as muscimol (Faure et al., 2010; Ho and Berridge, 2014). The GABA drug probably produces a stronger electrophysiological inhibition of shell neurons than the glutamate blocking drug (e.g., DNQX), which produces only a mode of pure coping ‘fear.’ Thus conceivably this incremental change in neural inhibitory signal produces a qualitative change in psychological emotion that adds a categorically new ‘disgust’ reaction to ‘fear’ reactions. Because nucleus accumbens neurons normally inhibit their targets (by themselves releasing GABA as neurotransmitter), this stronger inhibition could release downstream neurons in ventral pallidum and other targets into higher excitation levels, to produce the additional emotional reaction. Of course, an incremental difference at one overlapping stage of a neural circuit can still be translated into qualitative neural and psychological differences at other stages to recruit different anatomical patterns of activity. But exactly how that is accomplished remains to be understood.

Would similar neuronal manipulations of human nucleus accumbens neurons induce subjective fear versus disgust motivations in people too? We don’t yet know, though these are clear predictions from this schematic understanding of ‘fear’ and ‘disgust’ mechanisms. Someday, less invasive techniques of brain modulation, or more sensitive techniques of brain measurement, may allow such predictions to be tested in humans. People may turn out to have desire-dread keyboards in nucleus accumbens similar to those already found in rats that produce core ‘wanting’ and ‘liking’ versus ‘fear’ and ‘disgust.’ If so, such findings would indicate another continuity between human and animal mechanisms of emotion. Just as when the original dopamine-based discovery that ‘wanting’ versus ‘liking’ mechanisms were different in rats pointed the way to teasing apart subjective wanting and liking feelings in humans, conclusions first gained from objective affective reactions in animals can sometimes give us new and important insights into human emotions. In such cases, animal findings at the very least tell investigators what to look for in people, shaping the questions to be asked so that the right answers are found.

## Conclusion

Emotional and motivational processes occur in people most notably as subjective feelings, but also have underlying core psychological processes with objective features. These core affective processes can be schematically understood, so that their psychological features become objectively identified, and allowing them to be mapped to underlying brain mechanisms. In many cases, conclusions can transfer from affective neuroscience studies based on animals to human emotions and motivations. Admittedly, not all conclusions will transfer. But those that do often carry important implications for understanding human clinical affective disorders. Best progress in building an integrated psychology and affective neuroscience of emotion and motivation requires combining human and animal approaches together, and consideration of both subjective and objective features of affective reactions.

## Author Contributions

The author confirms being the sole contributor of this work, wrote the article and approved it for publication.

## Conflict of Interest Statement

The author declares that the research was conducted in the absence of any commercial or financial relationships that could be construed as a potential conflict of interest.

## References

[B1] Adolphs R. Gosselin F. Buchanan T. W. Tranel D. Schyns P. Damasio A. R. (2005). A mechanism for impaired fear recognition after amygdala damage. *Nature* 433 68–72. 10.1038/nature03086 15635411

[B2] Allen W. E. DeNardo L. A. Chen M. Z. Liu C. D. Loh K. M. Fenno L. E. (2017). Thirst-associated preoptic neurons encode an aversive motivational drive. *Science* 357 1149–1155. 10.1126/science.aan6747 28912243 PMC5723384

[B3] Anderson A. K. Yamaguchi Y. Grabski W. Lacka D. (2006). Emotional memories are not all created equal: evidence for selective memory enhancement. *Learn. Mem.* 13 711–718. 10.1101/lm.388906 17101871 PMC1783624

[B4] Anderson D. J. Adolphs R. (2014). A framework for studying emotions across species. *Cell* 157 187–200. 10.1016/j.cell.2014.03.003 24679535 PMC4098837

[B5] Arnold M. B. (1960). *Emotion and Personality.* New York, NY: Columbia University Press.

[B6] Barch D. M. Treadway M. T. Schoen N. (2014). Effort, anhedonia, and function in schizophrenia: reduced effort allocation predicts amotivation and functional impairment. *J. Abnorm. Psychol.* 123 387–397. 10.1037/a0036299 24886012 PMC4048870

[B7] Barrett L. F. (2017). *How Emotions are Made: The Secret Life of the Brain.* Boston, MA: Houghton Mifflin Harcourt.

[B8] Beaver J. D. Lawrence A. D. Passamonti L. Calder A. J. (2008). Appetitive motivation predicts the neural response to facial signals of aggression. *J. Neurosci.* 28 2719–2725. 10.1523/JNEUROSCI.0033-08.2008 18337401 PMC6670667

[B9] Berridge K. C. (2000). Measuring hedonic impact in animals and infants: microstructure of affective taste reactivity patterns. *Neurosci. Biobehav. Rev.* 24 173–198. 10.1016/S0149-7634(99)00072-X 10714382

[B10] Berridge K. C. (2001). “Reward learning: reinforcement, incentives, and expectations,” in *The Psychology of Learning and Motivation*, ed. Ross B. H. (Cambridge, MA: Academic Press), 223–278.

[B11] Berridge K. C. (2004). Motivation concepts in behavioral neuroscience. *Physiol. Behav.* 81 179–209. 10.1016/j.physbeh.2004.02.004 15159167

[B12] Berridge K. C. (2012). From prediction error to incentive salience: mesolimbic computation of reward motivation. *Eur. J. Neurosci.* 35 1124–1143. 10.1111/j.1460-9568.2012.07990.x 22487042 PMC3325516

[B13] Berridge K. C. Kringelbach M. L. (2015). Pleasure systems in the brain. *Neuron* 86 646–664. 10.1016/j.neuron.2015.02.018 25950633 PMC4425246

[B14] Berridge K. C. Robinson T. E. (2016). Liking, wanting, and the incentive-sensitization theory of addiction. *Am. Psychol.* 71 670–679. 10.1037/amp0000059 27977239 PMC5171207

[B15] Berridge K. C. Valenstein E. S. (1991). What psychological process mediates feeding evoked by electrical stimulation of the lateral hypothalamus? *Behav. Neurosci.* 105 3–14. 2025391 10.1037//0735-7044.105.1.3

[B16] Berridge K. C. Venier I. L. Robinson T. E. (1989). Taste reactivity analysis of 6-hydroxydopamine-induced aphagia: Implications for arousal and anhedonia hypotheses of dopamine function. *Behav. Neurosci.* 103 36–45. 10.1037/0735-7044.103.1.36 2493791

[B17] Berridge K. C. Winkielman P. (2003). What is an unconscious emotion? (The case for unconscious “liking”). *Cogn. Emot.* 17 181–211. 10.1080/02699930302289 29715719

[B18] Betley J. N. Xu S. Cao Z. F. Gong R. Magnus C. J. Yu Y. (2015). Neurons for hunger and thirst transmit a negative-valence teaching signal. *Nature* 521 180–185. 10.1038/nature14416 25915020 PMC4567040

[B19] Bindra D. (1978). How adaptive behavior is produced: a perceptual-motivation alternative to response reinforcement. *Behav. Brain Sci.* 1 41–91. 10.1017/S0140525X00059380

[B20] Boileau I. Dagher A. Leyton M. Gunn R. N. Baker G. B. Diksic M. (2006). Modeling sensitization to stimulants in humans: an [11C]raclopride/positron emission tomography study in healthy men. *Arch. Gen. Psychiatry* 63 1386–1395. 10.1001/archpsyc.63.12.1386 17146013

[B21] Bourin M. (2015). Animal models for screening anxiolytic-like drugs: a perspective. *Dialogues Clin. Neurosci.* 17 295–303. 26487810 10.31887/DCNS.2015.17.3/mbourin PMC4610614

[B22] Brauer L. H. De Wit H. (1997). High dose pimozide does not block amphetamine-induced euphoria in normal volunteers. *Pharmacol. Biochem. Behav.* 56 265–272. 10.1016/S0091-3057(96)00240-7 9050084

[B23] Cabanac M. (1971). Physiological role of pleasure. *Science* 173 1103–1107. 10.1126/science.173.4002.1103 5098954

[B24] Cabanac M. (1992). Pleasure: the common currency. *J. Theor. Biol.* 155 173–200. 10.1016/S0022-5193(05)80594-6 12240693

[B25] Calder A. Beaver J. Davis M. van Ditzhuijzen J. Keane J. Lawrence A. (2007). Disgust sensitivity predicts the insula and pallidal response to pictures of disgusting foods. *Eur. J. Neurosci.* 25 3422–3428. 10.1111/j.1460-9568.2007.05604.x 17553011

[B26] Callesen M. B. Scheel-Kruger J. Kringelbach M. L. Moller A. (2013). A systematic review of impulse control disorders in Parkinson’s disease. *J. Parkinsons Dis.* 3 105–138. 10.3233/JPD-120165 23938342

[B27] Campbell B. A. (1960). Effects of water deprivation on random activity. *J. Comp. Physiol. Psychol.* 53 240–241. 10.1037/h0044975 13807228

[B28] Carver C. S. (2009). Threat sensitivity, incentive sensitivity, and the experience of relief. *J. Pers.* 77 125–138. 10.1111/j.1467-6494.2008.00540.x 19076994

[B29] Castro D. C. Berridge K. C. (2017). Opioid and orexin hedonic hotspots in rat orbitofrontal cortex and insula. *Proc. Natl. Acad. Sci. U.S.A.* 114 E9125–E9134. 10.1073/pnas.1705753114 29073109 PMC5664503

[B30] Chapman H. Kim D. Susskind J. Anderson A. (2009). In bad taste: evidence for the oral origins of moral disgust. *Science* 323 1222–1226. 10.1126/science.1165565 19251631

[B31] Chapman H. A. Anderson A. K. (2012). Understanding disgust. *Ann. N. Y. Acad. Sci.* 1251 62–76. 10.1111/j.1749-6632.2011.06369.x 22256964

[B32] Chen Y. Lin Y.-C. Zimmerman C. A. Essner R. A. Knight Z. A. (2016). Hunger neurons drive feeding through a sustained, positive reinforcement signal. *eLife* 5:e18640. 10.7554/eLife.18640 27554486 PMC5016090

[B33] Clore G. L. (1994). “Why emotions are never unconscious,” in *The Nature of Emotion: Fundamental Questions*, eds Ekman P. Davidson R. J. (New York, NY: Oxford University Press), 285–290.

[B34] Clore G. L. Ortony A. (1984). Some issues for a cognitive theory of emotion. *Cah. Psychol. Cogn. Curr. Psychol. Cogn.* 4 53–57.

[B35] Cogan E. S. Shapses M. A. Robinson T. E. Tronson N. C. (2018). Disrupting reconsolidation: memory erasure or blunting of emotional/motivational value? *Neuropsychopharmacology* 10.1038/s41386-018-0082-0 [Epub ahead of print]. 29786067 PMC6300536

[B36] Coss R. G. Owings D. H. (1978). Snake-directed behavior by snake naive and experienced california ground squirrels in a simulated burrow. *Z. Tierpsychol. J. Comp. Ethol.* 48 421–435.

[B37] Damasio A. (2018). *The Strange Order of Things: Life, feeling, and the making of cultures.* New York, NY: Pantheon books.

[B38] Damasio A. R. (2004). “Emotions and feelings: a neurobiological perspective,” in *Feelings and Emotions: The Amsterdam Symposium*, ed. Manstead A. S. R. (Cambridge: Cambridge University Press), 49–57. 10.1017/CBO9780511806582.004

[B39] Darwin C. (1872). *The Expression of the Emotions in Man and Animals (1998 edition: revised and with commentary by P. Ekman).* Oxford: Oxford University Press 10.1037/10001-000

[B40] Davis C. Carter J. C. (2009). Compulsive overeating as an addiction disorder. A review of theory and evidence. *Appetite* 53 1–8. 10.1016/j.appet.2009.05.018 19500625

[B41] Dayan P. Berridge K. C. (2014). Model-based and model-free Pavlovian reward learning: revaluation, revision, and revelation. *Cogn. Affect. Behav. Neurosci* 14 473–492. 10.3758/s13415-014-0277-8 24647659 PMC4074442

[B42] Dickinson A. Balleine B. (2002). “The role of learning in the operation of motivational systems,” in *Stevens’ Handbook of Experimental Psychology: Learning, Motivation, and Emotion*, 3 Edn, ed. Gallistel C. R. (New York: Wiley and Sons), 497–534.

[B43] Dickinson A. Balleine B. (2010). “Hedonics: The Cognitive-Motivational Interface,” in *Pleasures of the Brain*, eds Kringelbach M. L. Berridge K. C. (Oxford: Oxford University Press), 74–84.

[B44] Dijksterhuis A. Bos M. W. Nordgren L. F. van Baaren R. B. (2006). On making the right choice: the deliberation-without-attention effect. *Science* 311 1005–1007. 10.1126/science.1121629 16484496

[B45] Dowd E. C. Barch D. M. (2010). Anhedonia and emotional experience in schizophrenia: neural and behavioral indicators. *Biol. Psychiatry* 67 902–911. 10.1016/j.biopsych.2009.10.020 20004364 PMC3113677

[B46] Ekman P. (1999). “Basic emotions,” in *Handbook of Cognition and Emotion*, eds Dalgleish T. Power M. (Chichester: John Wiley & Sons Ltd), 45–60.

[B47] Ellsworth P. C. (1994). “Levels of thought and levels of emotion,” in *The Nature of Emotion: Fundamental Questions*, eds Ekman P. Davidson R. J. (New York, NY: Oxford University Press), 192–196.

[B48] Ellsworth P. C. Scherer K. R. (2003). “Appraisal processes in emotion,” in *Handbook of Affective Sciences*, eds Davidson R. J. Sherer K. R. Goldsmith H. H. (Oxford: Oxford University Press), 572–595.

[B49] Evans A. H. Pavese N. Lawrence A. D. Tai Y. F. Appel S. Doder M. (2006). Compulsive drug use linked to sensitized ventral striatal dopamine transmission. *Ann. Neurol.* 59 852–858. 10.1002/ana.20822 16557571

[B50] Faure A. Richard J. M. Berridge K. C. (2010). Desire and dread from the nucleus accumbens: cortical glutamate and subcortical GABA differentially generate motivation and hedonic impact in the rat. *PLoS One* 5:e11223. 10.1371/journal.pone.0011223 20585461 PMC2887893

[B51] Fischman M. W. Foltin R. W. (1992). *Self-Administration of Cocaine by Humans: a Laboratory Perspective, Cocaine: Scientific and Social Dimensions, CIBA foundation symposium No 166*, (Chichester: Wiley),165–180. 10.1002/9780470514245.ch10 1638911

[B52] Flagel S. B. Robinson T. E. (2017). Neurobiological basis of individual variation in stimulus-reward learning. *Curr. Opin. Behav. Sci.* 13 178–185. 10.1016/j.cobeha.2016.12.004 28670608 PMC5486979

[B53] Freud S. (1950). “Collected papers,” in *International Psycho-Analytical Library*, ed. Freeman S. (London: Hogarth Press), 508.

[B54] Friedman J. H. Chang V. (2013). Crack cocaine use due to dopamine agonist therapy in Parkinson disease. *Neurology* 80 2269–2270. 10.1212/WNL.0b013e318296e9d5 23677745 PMC3721099

[B55] Frijda N. H. (2007). *Universal The Laws of Emotion.* Mahwah, NJ: Lawrence Erlbaum Associates.

[B56] Frijda N. H. (2016). The evolutionary emergence of what we call “emotions”. *Cogn. Emot.* 30 609–620. 10.1080/02699931.2016.1145106 26943795

[B57] Frijda N. H. Parrott W. G. (2011). Basic emotions or ur-emotions? *Emot. Rev.* 3 406–415. 10.1177/1754073911410742

[B58] Gearhardt A. N. Yokum S. Orr P. T. Stice E. Corbin W. R. Brownell K. D. (2011). Neural correlates of food addiction. *Arch. Gen. Psychiatry* 68 808–816. 10.1001/archgenpsychiatry.2011.32 21464344 PMC3980851

[B59] Gray J. A. Kumari V. Lawrence N. Young A. M. J. (1999). Functions of the dopaminergic innervation of the nucleus accumbens. *Psychobiology* 27 225–235.

[B60] Greenwald A. G. Banaji M. R. (2017). The implicit revolution: reconceiving the relation between conscious and unconscious. *Am. Psychol.* 72 861–871. 10.1037/amp0000238 29283625

[B61] Grill H. J. Norgren R. (1978). The taste reactivity test. I. Mimetic responses to gustatory stimuli in neurologically normal rats. *Brain Res.* 143 263–279. 10.1016/0006-8993(78)90568-1 630409

[B62] Hartston H. (2012). The case for compulsive shopping as an addiction. *J. Psychoactive Drugs* 44 64–67. 10.1080/02791072.2012.660110 22641966

[B63] Herzog J. Reiff J. Krack P. Witt K. Schrader B. Muller D. (2003). Manic episode with psychotic symptoms induced by subthalamic nucleus stimulation in a patient with Parkinson’s disease. *Mov. Disord.* 18 1382–1384. 10.1002/mds.10530 14639687

[B64] Hickey C. Peelen M. V. (2015). Neural mechanisms of incentive salience in naturalistic human vision. *Neuron* 85 512–518. 10.1016/j.neuron.2014.12.049 25654257

[B65] Higgins E. T. (2006). Value from hedonic experience and engagement. *Psychol. Rev.* 113 439–460. 10.1037/0033-295X.113.3.439 16802877

[B66] Ho C.-Y. Berridge K. C. (2014). Excessive disgust caused by brain lesions or temporary inactivations: mapping hotspots of the nucleus accumbens and ventral pallidum. *Eur. J. Neurosci.* 40 3556–3572. 10.1111/ejn.12720 25229197 PMC4236281

[B67] Holland P. C. Petrovich G. D. Gallagher M. (2002). The effects of amygdala lesions on conditioned stimulus- potentiated eating in rats. *Physiol. Behav.* 76 117–129. 10.1016/S0031-9384(02)00688-1 12175595

[B68] Holman G. L. (1969). Intragastric reinforcement effect. *J. Comp. Physiol. Psychol.* 69 432–441. 10.1037/h0028233 5349026

[B69] Howes O. D. Kapur S. (2009). The dopamine hypothesis of schizophrenia: version III–the final common pathway. *Schizophr. Bull.* 35 549–562. 10.1093/schbul/sbp006 19325164 PMC2669582

[B70] Hull C. L. (1951). *Essentials of Behavior.* New Haven: Yale University Press.

[B71] Huston J. P. (1972). “Classical conditioning of consummatory behavior,” in *Control Mechanisms of Drinking*, eds Peters G. Fitzsimons J. T. Peters-Haefeli L. (Berlin: Springer-Verlag), 165–172.

[B72] James W. (1884). What is an emotion. *Mind* 9 188–205. 10.1093/mind/os-IX.34.188

[B73] James W. (1902). *The Varieties of Religious Experience: A Study in Human Nature.* New York: Longmans, Green & Co.

[B74] James W. (1920). “Letter on Happiness to Miss Frances R. Morse (1901),” in *Letters of William James*, ed. James H. (Boston: Atlantic Monthly Press).

[B75] Jauregui-Lobera I. Bolanos-Rios P. Valero E. Ruiz Prieto I. (2012). Induction of food craving experience: the role of mental imagery, dietary restraint, mood and coping strategies. *Nutr. Hosp.* 27 1928–1935. 10.3305/nh.2012.27.6.6043 23588440

[B76] Keltner D. Ekman P. (2000). “Facial expression of emotion,” in *Handbook of emotions*, eds Lewis M. Haviland-Jones J. M. (New York, NY: Guilford), 236–249.

[B77] Keramati M. Durand A. Girardeau P. Gutkin B. Ahmed S. H. (2017). Cocaine addiction as a homeostatic reinforcement learning disorder. *Psychol. Rev.* 124 130–153. 10.1037/rev0000046 28095003

[B78] Kochel A. Plichta M. M. Schafer A. Leutgeb V. Scharmuller W. Fallgatter A. J. (2011). Affective perception and imagery: a NIRS study. *Int. J. Psychophysiol.* 80 192–197. 10.1016/j.ijpsycho.2011.03.006 21419180

[B79] Koob G. F. Volkow N. D. (2016). Neurobiology of addiction: a neurocircuitry analysis. *Lancet Psychiatry* 3 760–773. 10.1016/S2215-0366(16)00104-8 27475769 PMC6135092

[B80] Kruglanski A. W. Chernikova M. Rosenzweig E. Kopetz C. (2014). On motivational readiness. *Psychol. Rev.* 121 367–388. 10.1037/a0037013 25090424

[B81] Lazarus R. S. (1981). A cognitivist’s reply to Zajonc on emotion and cognition. *Am. Psychol.* 36 222–223. 10.1037/0003-066X.36.2.222

[B82] LeDoux J. (1996). *The Emotional Brain: The Mysterious Underpinnings of Emotional Life.* New York: Simon & Schuster.

[B83] LeDoux J. (2015). *Anxious: Using the Brain to Understand and Treat Fear and Anxiety.* New York, NY: Penguin Random House.

[B84] LeDoux J. E. (2014). Coming to terms with fear. *Proc. Natl. Acad. Sci. U.S.A.* 111 2871–2878. 10.1073/pnas.1400335111 24501122 PMC3939902

[B85] LeDoux J. E. (2015). Feelings: What Are They & How Does the Brain Make Them? *Daedalus-Us.* 144 96–111. 10.1162/DAED_a_00319

[B86] LeDoux J. E. Hofmann S. G. (2018). The subjective experience of emotion: a fearful view. *Curr. Opin. Behav. Sci.* 19 67–72. 10.1016/j.cobeha.2017.09.011 PMC11444666 39355512

[B87] Leshem M. (2009). Biobehavior of the human love of salt. *Neurosci. Biobehav. Rev.* 33 1–17. 10.1016/j.neubiorev.2008.07.007 18708089

[B88] Leyton M. (2010). “The neurobiology of desire: dopamine and the regulation of mood and motivational states in humans,” in *Pleasures of the Brain*, eds Kringelbach M. L. Berridge K. C. (Oxford: Oxford University Press), 222–243.

[B89] Leyton M. Boileau I. Benkelfat C. Diksic M. Baker G. Dagher A. (2002). Amphetamine-Induced Increases in extracellular dopamine, drug wanting, and novelty seeking: a PET/[11C]raclopride study in healthy men. *Neuropsychopharmacology* 27 1027–1035. 10.1016/S0893-133X(02)00366-4 12464459

[B90] Li X. Zeric T. Kambhampati S. Bossert J. M. Shaham Y. (2015). The central amygdala nucleus is critical for incubation of methamphetamine craving. *Neuropsychopharmacology* 40 1297–1306. 10.1038/npp.2014.320 25475163 PMC4367476

[B91] Liggins J. Pihl R. O. Benkelfat C. Leyton M. (2012). The dopamine augmenter L-DOPA does not affect positive mood in healthy human volunteers. *PLoS One* 7:e28370. 10.1371/journal.pone.0028370 22238577 PMC3251561

[B92] Linnet J. Mouridsen K. Peterson E. Møller A. Doudet D. J. Gjedde A. (2012). Striatal dopamine release codes uncertainty in pathological gambling. *Psychiatry Res. Neuroimaging* 204 55–60. 10.1016/j.pscychresns.2012.04.012 22889563

[B93] Maren S. Phan K. L. Liberzon I. (2013). The contextual brain: implications for fear conditioning, extinction and psychopathology. *Nat. Rev. Neurosci.* 14 417–428. 10.1038/nrn3492 23635870 PMC5072129

[B94] Mason W. A. Capitanio J. P. (2012). Basic emotions: a reconstruction. *Emot. Rev.* 4 238–244. 10.1177/1754073912439763 27110280 PMC4840933

[B95] May J. Andrade J. Panabokke N. Kavanagh D. (2004). Images of desire: cognitive models of craving. *Memory* 12 447–461. 10.1080/09658210444000061 15493072

[B96] Miller N. E. (1971). *Neal E. Miller: Selected Papers.* Chicago: Aldine Atherton.

[B97] Miller N. E. (1973). “How the project started,” in *Brain Stimulation and Motivation: Research and Commentary*, ed. Valenstein E. S. (Glenview, IL: Scott, Foresman and company), 53–68.

[B98] Miller N. E. Kessen M. L. (1952). Reward effects of food via stomach fistula compared with those of food via mouth. *J. Comp. Physiol. Psychol.* 45 555–564. 10.1037/h0060113 13000028

[B99] Mineka S. Seligman M. E. Hetrick M. Zuelzer K. (1972). Poisoning and conditioned drinking. *J. Comp. Physiol. Psychol.* 79 377–384. 10.1037/h0032825 5054474

[B100] Mischel W. Shoda Y. Rodriguez M. I. (1989). Delay of gratification in children. *Science* 244 933–938. 10.1126/science.2658056 2658056

[B101] Miyapuram K. P. Tobler P. N. Gregorios-Pippas L. Schultz W. (2012). BOLD responses in reward regions to hypothetical and imaginary monetary rewards. *Neuroimage* 59 1692–1699. 10.1016/j.neuroimage.2011.09.029 21985912 PMC4290830

[B102] Moan C. E. Heath R. G. (1972). Septal stimulation for initiation of heterosexual behavior in a homosexual male. *J. Behav. Ther. Exp. Psychiatry* 3 23–30. 10.1016/0005-7916(72)90029-8

[B103] Morris J. S. Ohman A. Dolan R. J. (1998). Conscious and unconscious emotional learning in the human amygdala. *Nature* 393 467–470. 10.1038/30976 9624001

[B104] Nagel T. (1974). What is it like to be a bat. *Philos. Rev.* 83 435–450. 10.2307/2183914

[B105] Nicolaidis S. Rowland N. (1975). Regulatory drinking in rats with permanent access to a bitter fluid source. *Physiol. Behav.* 14 819–824. 10.1016/0031-9384(75)90076-1 1187838

[B106] Nisbett R. W. Wilson T. D. (1978). Telling more than we can know: verbal reports on mental processes. *Psychol. Rev.* 84 231–259. 10.1037/0033-295X.84.3.231 17049881

[B107] Olds J. (1973). “The discovery of reward systems in the brain,” in *Brain Stimulation and Motivation: research and commentary*, ed. Valenstein E. S. (Glenview, Il: Scott, Foresman and company), 81–99.

[B108] Olds J. Milner P. (1954). Positive reinforcement produced by electrical stimulation of septal area and other regions of rat brain. *J. Comp. Physiol. Psychol.* 47 419–427. 10.1037/h0058775 13233369

[B109] Ondo W. G. Lai D. (2008). Predictors of impulsivity and reward seeking behavior with dopamine agonists. *Parkinsonism Relat. Disord.* 14 28–32. 10.1016/j.parkreldis.2007.05.006 17702628

[B110] O’Sullivan S. Evans A. Lees A. (2009). Dopamine dysregulation syndrome: an overview of its epidemiology, mechanisms and management. *CNS Drugs* 23 157–170. 10.2165/00023210-200923020-00005 19173374

[B111] O’Sullivan S. S. Wu K. Politis M. Lawrence A. D. Evans A. H. Bose S. K. (2011). Cue-induced striatal dopamine release in Parkinson’s disease-associated impulsive-compulsive behaviours. *Brain* 134 969–978. 10.1093/brain/awr003 21349901

[B112] Panksepp J. (1986). The neurochemistry of behavior. *Annu. Rev. Psychol.* 37 77–107. 10.1146/annurev.ps.37.020186.000453 3963784

[B113] Panksepp J. (2011). The basic emotional circuits of mammalian brains: do animals have affective lives? *Neurosci. Biobehav. Rev.* 35 1791–1804. 10.1016/j.neubiorev.2011.08.003 21872619

[B114] Paré D. Quirk G. J. (2017). When scientific paradigms lead to tunnel vision: lessons from the study of fear. *Sci. Learn.* 2:6 10.1038/s41539-017-0007-4 PMC6171770 30294453

[B115] Paulson P. E. Camp D. M. Robinson T. E. (1991). Time course of transient behavioral depression and persistent behavioral sensitization in relation to regional brain monoamine concentrations during amphetamine withdrawal in rats. *Psychopharmacology* 103 480–492. 10.1007/BF02244248 2062986 PMC1865099

[B116] Pecina S. Berridge K. C. (2013). Dopamine or opioid stimulation of nucleus accumbens similarly amplify cue-triggered ’wanting’ for reward: entire core and medial shell mapped as substrates for PIT enhancement. *Eur. J. Neurosci.* 37 1529–1540. 10.1111/ejn.12174 23495790 PMC4028374

[B117] Peciña S. Cagniard B. Berridge K. C. Aldridge J. W. Zhuang X. (2003). Hyperdopaminergic mutant mice have higher “wanting” but not “liking” for sweet rewards. *J. Neurosci.* 23 9395–9402. 10.1523/JNEUROSCI.23-28-09395.2003 14561867 PMC6740586

[B118] Perusini J. N. Fanselow M. S. (2015). Neurobehavioral perspectives on the distinction between fear and anxiety. *Learn. Mem.* 22 417–425. 10.1101/lm.039180.115 26286652 PMC4561408

[B119] Petrovich G. D. (2011). Learning and the motivation to eat: forebrain circuitry. *Physiol. Behav.* 104 582–589. 10.1016/j.physbeh.2011.04.059 21549730 PMC3446257

[B120] Politis M. Loane C. Wu K. O’Sullivan S. S. Woodhead Z. Kiferle L. (2013). Neural response to visual sexual cues in dopamine treatment-linked hypersexuality in Parkinson’s disease. *Brain* 136 400–411. 10.1093/brain/aws326 23378222

[B121] Popper K. R. (1972). *The Logic of Scientific Discovery. Impression Revised*, 6th Edn. London: Hutchinson.

[B122] Ray N. Miyasaki J. M. Zurowski M. Ko J. H. Cho S. S. Pellecchia G. (2012). Extrastriatal dopaminergic abnormalities of DA homeostasis in Parkinson’s patients with medication-induced pathological gambling: a [11C] FLB-457 and PET study. *Neurobiol. Dis.* 48 519–525. 10.1016/j.nbd.2012.06.021 22766031 PMC3465363

[B123] Reynolds S. M. Berridge K. C. (2008). Emotional environments retune the valence of appetitive versus fearful functions in nucleus accumbens. *Nat. Neurosci.* 11 423–425. 10.1038/nn2061 18344996 PMC2717027

[B124] Richard J. M. Berridge K. C. (2011). Nucleus accumbens dopamine/glutamate interaction switches modes to generate desire versus dread: D1 alone for appetitive eating but D1 and D2 together for fear. *J. Neurosci.* 31 12866–12879. 10.1523/JNEUROSCI.1339-11.2011 21900565 PMC3174486

[B125] Robinson M. J. Berridge K. C. (2013). Instant transformation of learned repulsion into motivational “wanting”. *Curr. Biol.* 23 282–289. 10.1016/j.cub.2013.01.016 23375893 PMC3580026

[B126] Robinson T. E. Berridge K. C. (1993). The neural basis of drug craving: an incentive-sensitization theory of addiction. *Brain Res. Brain Res. Rev.* 18 247–291. 10.1016/0165-0173(93)90013-P 8401595

[B127] Rozin P. Haidt J. McCauley C. R. (2008). “Disgust,” in *Handbook of Emotions*, 3rd Edn, eds Lewis M. Haviland J. M.-Jones (New York, NY: Guilford Publications), 757–776.

[B128] Russell J. A. Barrett L. F. (1999). Core affect, prototypical emotional episodes, and other things called emotion: dissecting the elephant. *J. Pers. Soc. Psychol.* 76 805–819. 10.1037/0022-3514.76.5.805 10353204

[B129] Ryan R. M. Deci E. L. (2000). Self-determination theory and the facilitation of intrinsic motivation, social development, and well-being. *Am. Psychol.* 55 68–78. 10.1037/0003-066X.55.1.68 11392867

[B130] Salamone J. Pardo M. Yohn S. López-Cruz L. SanMiguel N. Correa M. (2015). *Mesolimbic Dopamine and the Regulation of Motivated Behavior.* Berlin: Springer, 1–27. 10.1007/7854_2015_383 26323245

[B131] Salamone J. D. (1991). “Behavioral pharmacology of dopamine systems: a new synthesis,” in *The Mesolimbic Dopamine System : From Motivation to Action*, ed. Willner P. (New York, NY: John Wiley & Sons), 599–611.

[B132] Salamone J. D. Correa M. Yang J. H. Rotolo R. Presby R. (2018). Dopamine, effort-based choice, and behavioral economics: basic and translational research. *Front. Behav. Neurosci.* 12:52. 10.3389/fnbeh.2018.00052 29628879 PMC5876251

[B133] Saunders B. T. Robinson T. E. (2013). Individual variation in resisting temptation: implications for addiction. *Neurosci. Biobehav. Rev.* 37 1955–1975. 10.1016/j.neubiorev.2013.02.008 23438893 PMC3732519

[B134] Schachter S. Singer J. E. (1962). Cognitive, social, and physiological determinants of emotional state. *Psychol. Rev.* 69 379–399. 10.1037/h0046234 14497895

[B135] Schlaepfer T. E. Cohen M. X. Frick C. Kosel M. Brodesser D. Axmacher N. (2008). Deep brain stimulation to reward circuitry alleviates anhedonia in refractory major depression. *Neuropsychopharmacology.* 33 368–377. 10.1038/sj.npp.1301408 17429407

[B136] Schooler J. W. Mauss I. B. (2010). “To be happy and to know it: The experience and meta-awareness of pleasure,” in *Pleasures of the Brain*, eds Kringelbach M. L. Berridge K. C. (Oxford: Oxford University Press), 244–254.

[B137] Schwarz N. (1999). Self-reports: how the questions shape the answers. *Am. Psychol.* 54 93–105. 10.1097/ACM.0000000000002002 29095172

[B138] Sienkiewicz-Jarosz H. Scinska A. Kuran W. Ryglewicz D. Rogowski A. Wrobel E. (2005). Taste responses in patients with Parkinson’s disease. *J. Neurol. Neurosurg. Psychiatry* 76 40–46. 10.1136/jnnp.2003.033373 15607993 PMC1739334

[B139] Sienkiewicz-Jarosz H. Scinska A. Swiecicki L. Lipczynska-Lojkowska W. Kuran W. Ryglewicz D. (2013). Sweet liking in patients with Parkinson’s disease. *J. Neurol. Sci.* 329 17–22. 10.1016/j.jns.2013.03.005 23561981

[B140] Smith K. S. Berridge K. C. (2007). Opioid limbic circuit for reward: interaction between hedonic hotspots of nucleus accumbens and ventral pallidum. *J. Neurosci.* 27 1594–1605. 10.1523/JNEUROSCI.4205-06.2007 17301168 PMC6673729

[B141] Smith K. S. Berridge K. C. Aldridge J. W. (2011). Disentangling pleasure from incentive salience and learning signals in brain reward circuitry. *Proc. Natl. Acad. Sci. U.S.A.* 108 E255–E264. 10.1073/pnas.1101920108 21670308 PMC3131314

[B142] Solomon R. L. Corbit J. D. (1974). An opponent-process theory of motivation. I. Temporal dynamics of affect. *Psychol. Rev.* 81 119–145. 10.1037/h0036128 4817611

[B143] Steiner J. E. Glaser D. Hawilo M. E. Berridge K. C. (2001). Comparative expression of hedonic impact: affective reactions to taste by human infants and other primates. *Neurosci. Biobehav. Rev.* 25 53–74. 10.1016/S0149-7634(00)00051-8 11166078

[B144] Strausfeld N. J. Hirth F. (2013). Deep homology of arthropod central complex and vertebrate Basal Ganglia. *Science* 340 157–161. 10.1126/science.1231828 23580521

[B145] Stuber G. D. Wise R. A. (2016). Lateral hypothalamic circuits for feeding and reward. *Nat. Neurosci.* 19 198–205. 10.1038/nn.4220 26814589 PMC4927193

[B146] Susskind J. M. Lee D. H. Cusi A. Feiman R. Grabski W. Anderson A. K. (2008). Expressing fear enhances sensory acquisition. *Nat. Neurosci.* 11 843–850. 10.1038/nn.2138 18552843

[B147] Swanson L. W. (2005). Anatomy of the soul as reflected in the cerebral hemispheres: neural circuits underlying voluntary control of basic motivated behaviors. *J. Comp. Neurol.* 493 122–131. 10.1002/cne.20733 16254987

[B148] Titchener E. B. (1902). *A Primer of Psychology. Rev.* London: The Macmillan company.

[B149] Toates F. (1986). *Motivational Systems.* Cambridge: Cambridge University Press.

[B150] Torre J. B. Lieberman M. D. (2018). Putting feelings into words: affect labeling as implicit emotion regulation. *Emot. Rev.* 10 116–124. 10.1177/1754073917742706

[B151] Treadway M. T. Zald D. H. (2011). Reconsidering anhedonia in depression: lessons from translational neuroscience. *Neurosci. Biobehav. Rev.* 35 537–555. 10.1016/j.neubiorev.2010.06.006 20603146 PMC3005986

[B152] Treit D. Berridge K. C. (1990). A comparison of benzodiazepine, serotonin, and dopamine agents in the taste-reactivity paradigm. *Pharmacol. Biochem. Behav.* 37 451–456. 10.1016/0091-3057(90)90011-6 1982355

[B153] Treit D. Engin E. McEown K. (2010). “Animal Models of Anxiety and Anxiolytic Drug Action,” in *Behavioral Neurobiology of Anxiety and Its Treatment*, eds Stein M. B. Steckler T. (Berlin: Springer), 121–160. 10.1007/7854_2009_17 21309109

[B154] Turner L. H. Solomon R. L. Stellar E. Wampler S. N. (1975). Humoral factors controlling food intake in dogs. *Acta Neurobiol. Exp.* 35 491–498. 1211243

[B155] Valenstein E. S. Cox V. C. Kakolewski J. W. (1970). Reexamination of the role of the hypothalamus in motivation. *Psychol. Rev.* 77 16–31. 10.1037/h0028581 4908030

[B156] Voon V. Mole T. B. Banca P. Porter L. Morris L. Mitchell S. (2014). Neural correlates of sexual cue reactivity in individuals with and without compulsive sexual behaviours. *PLoS One* 9:e102419. 10.1371/journal.pone.0102419 25013940 PMC4094516

[B157] Weingarten H. P. (1983). Conditioned cues elicit feeding in sated rats: a role for learning in meal initiation. *Science* 220 431–433. 10.1126/science.6836286 6836286

[B158] Weizenbaum J. (1977). *Computer Power and Human Reason: from Judgment to Calculation.* New York, NY: W.H. Freeman.

[B159] Wilkins L. Richter C. P. (1940). A great craving for salt by a child with cortico-adrenal insufficiency. *J. Am. Med. Assoc.* 114 866–868. 10.1001/jama.1940.62810100001011

[B160] Wilson T. D. Schooler J. W. (1991). Thinking too much: introspection can reduce the quality of preferences and decisions. *J. Pers. Soc. Psychol.* 60 181–192. 10.1037/0022-3514.60.2.181 2016668

[B161] Winkielman P. Berridge K. C. (2004). Unconscious emotion. *Curr. Dir. Psychol.* 13 120–123. 10.1111/j.0963-7214.2004.00288.x

[B162] Winkielman P. Berridge K. C. Wilbarger J. L. (2005). Unconscious affective reactions to masked happy versus angry faces influence consumption behavior and judgments of value. *Pers. Soc. Psychol. Bull.* 31 121–135. 10.1177/0146167204271309 15574667

[B163] Wise R. A. (1980). The dopamine synapse and the notion of ’pleasure centers’ in the brain. *Trends Neurosci.* 3 91–95. 10.1016/0166-2236(80)90035-1

[B164] Wyvell C. L. Berridge K. C. (2000). Intra-accumbens amphetamine increases the conditioned incentive salience of sucrose reward: enhancement of reward “wanting” without enhanced “liking” or response reinforcement. *J. Neurosci.* 20 8122–8130. 10.1523/JNEUROSCI.20-21-08122.2000 11050134 PMC6772712

[B165] Zajonc R. B. (1980). Feeling and thinking: preferences need no inferences. *Am. Psychol.* 35 151–175. 10.1037/0003-066X.35.2.151

